# Supplementary figures and images for: The olfactory receptor Olfr78 promotes differentiation of enterochromaffin cells in the mouse colon
Source: EMBO Rep. 2023 Dec 15;25(1):19. doi: 10.1038/s44319-023-00013-5 (PMC10897383; doi:10.1038/s44319-023-00013-5)

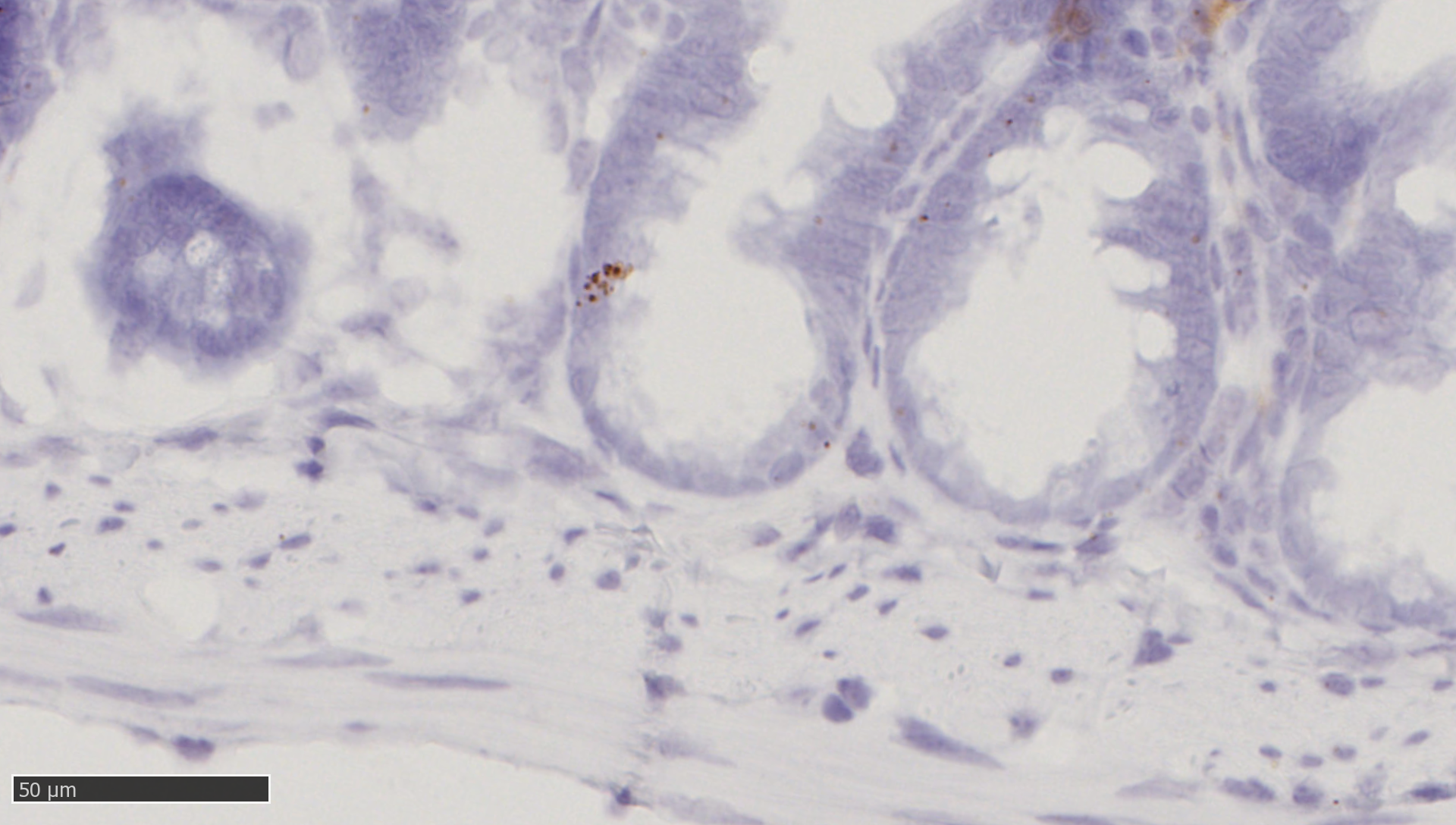

Supplement: Supplementary file 2 — Source Data Fig. 1 [file 44319_2023_13_MOESM2_ESM.zip › Figure 1/1B/1B Ileum Olfr558.tif]

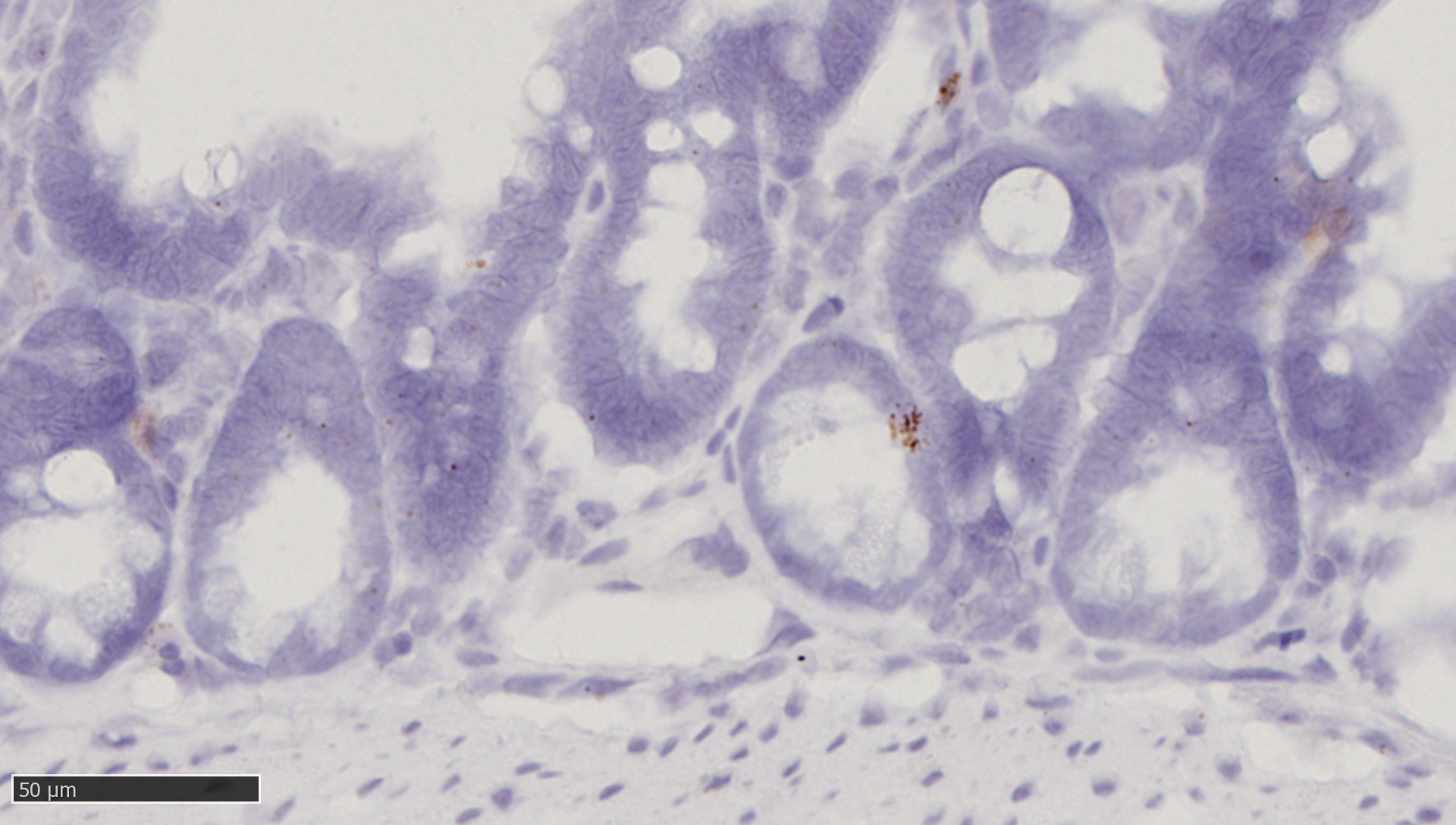

Supplement: Supplementary file 2 — Source Data Fig. 1 [file 44319_2023_13_MOESM2_ESM.zip › Figure 1/1B/1B Ileum Olfr78.tif]

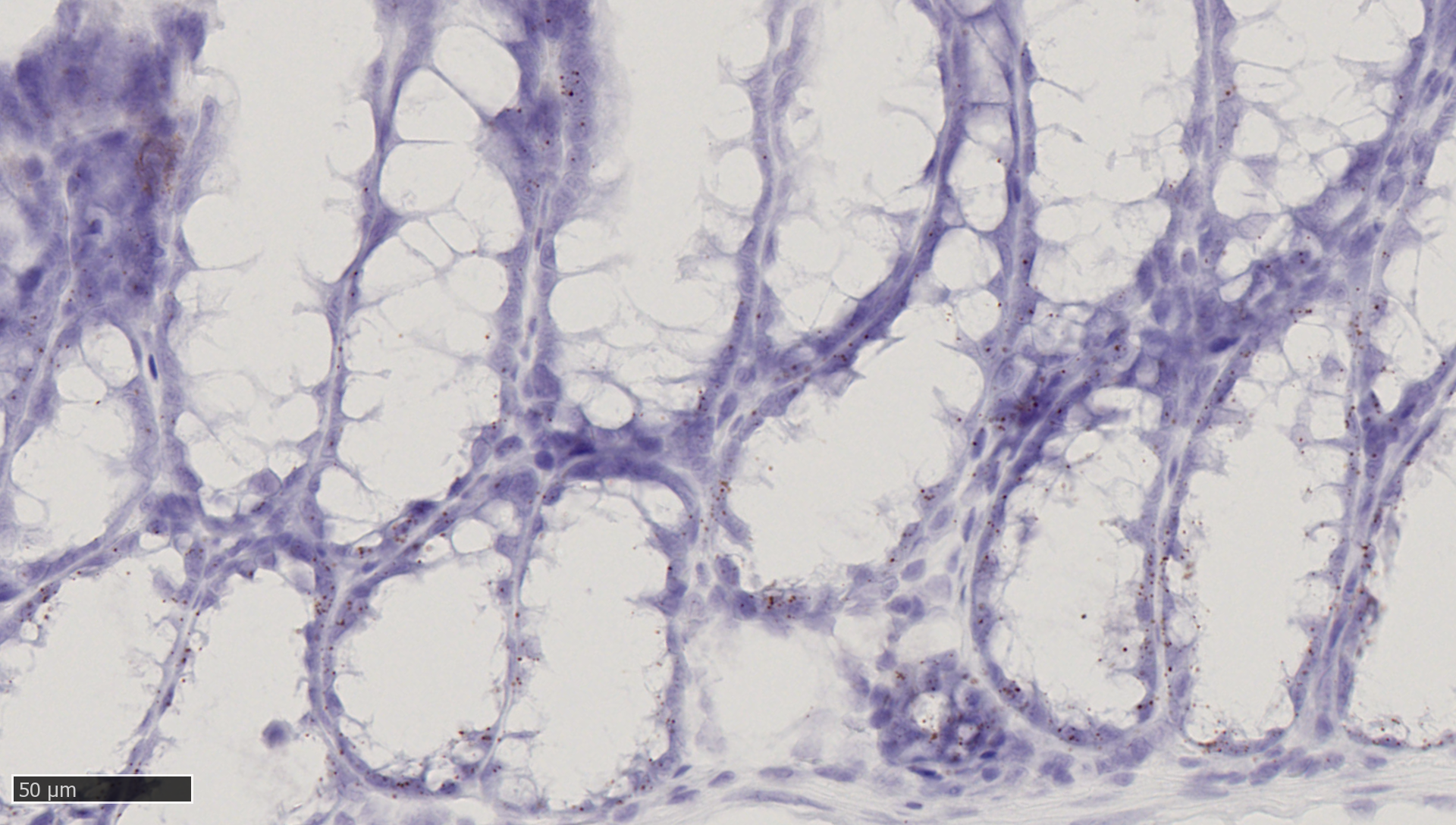

Supplement: Supplementary file 2 — Source Data Fig. 1 [file 44319_2023_13_MOESM2_ESM.zip › Figure 1/1B/1B Distal Colon Ffar2.tif]

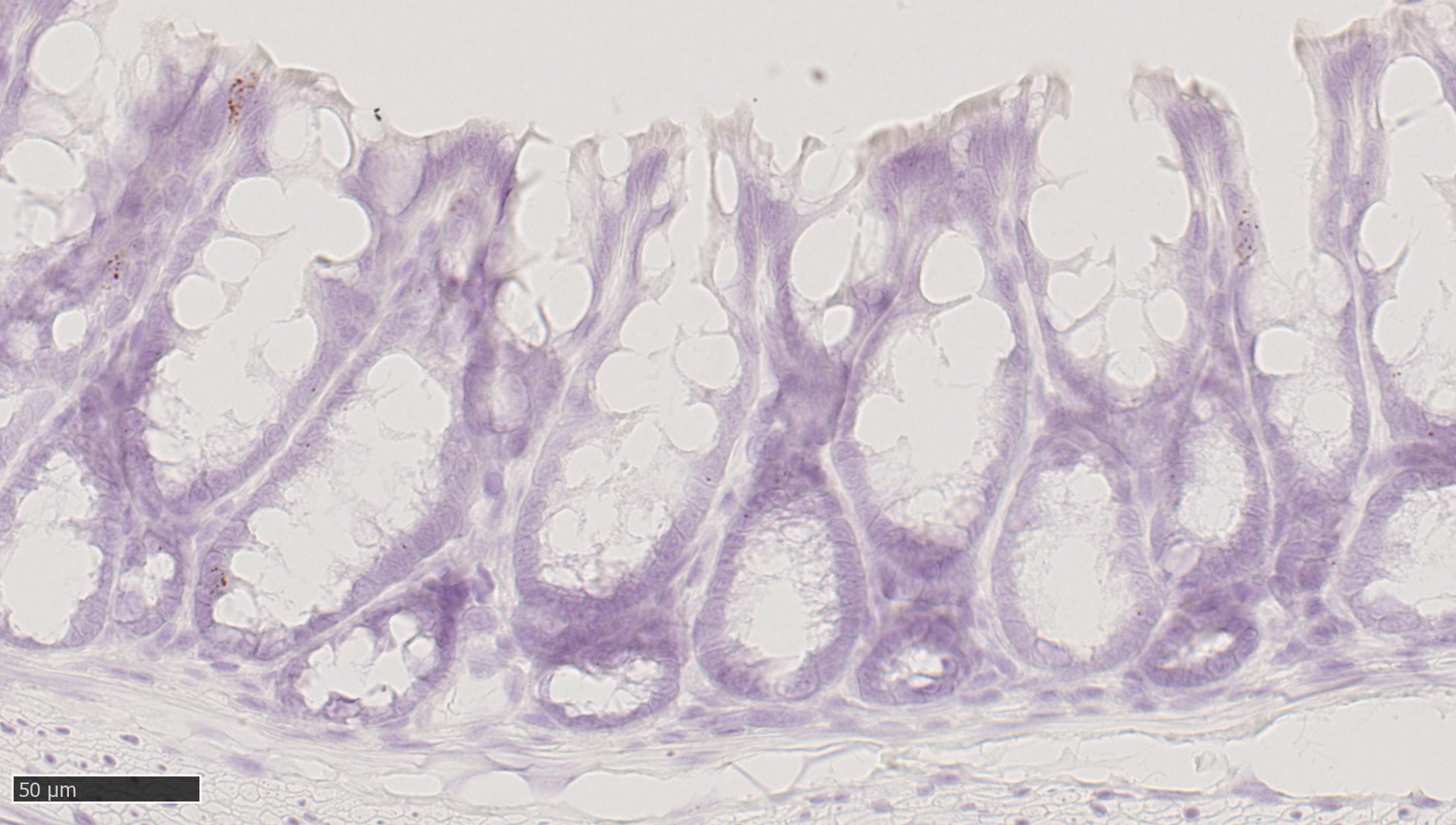

Supplement: Supplementary file 2 — Source Data Fig. 1 [file 44319_2023_13_MOESM2_ESM.zip › Figure 1/1B/1B Distal Colon Ffar3.tif]

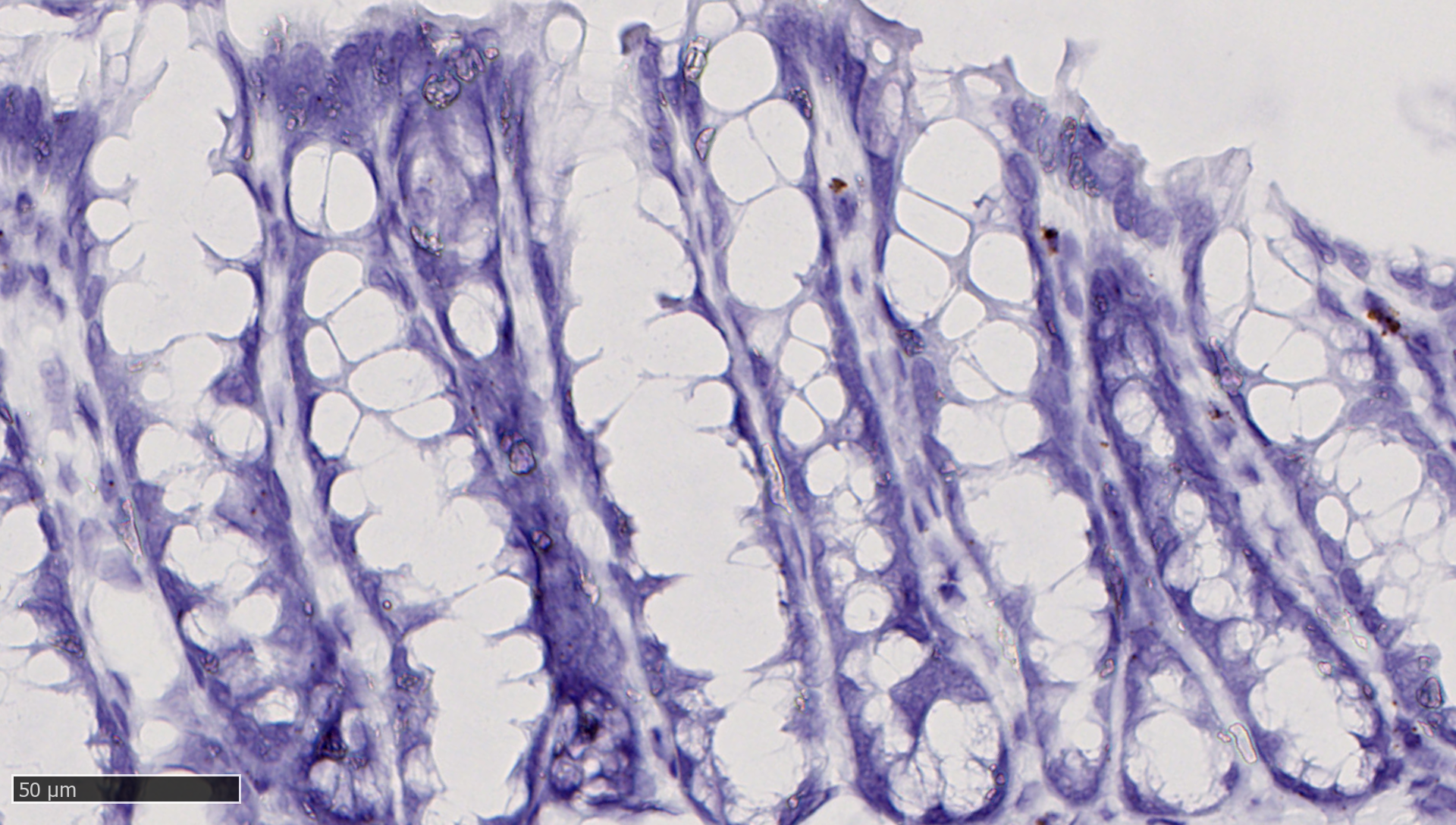

Supplement: Supplementary file 2 — Source Data Fig. 1 [file 44319_2023_13_MOESM2_ESM.zip › Figure 1/1B/1B Distal Colon Olfr558.tif]

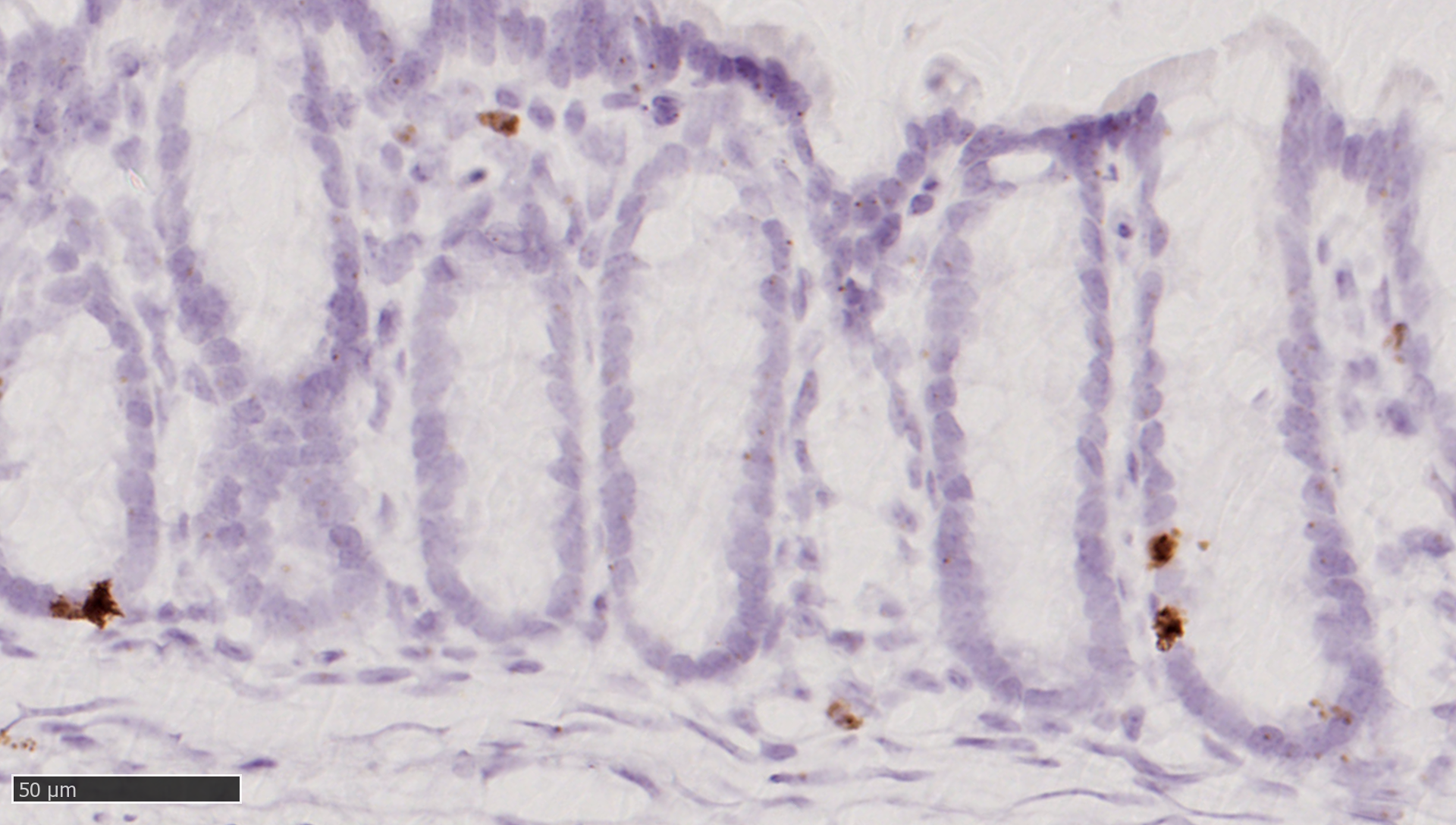

Supplement: Supplementary file 2 — Source Data Fig. 1 [file 44319_2023_13_MOESM2_ESM.zip › Figure 1/1B/1B Distal Colon Olfr78.tif]

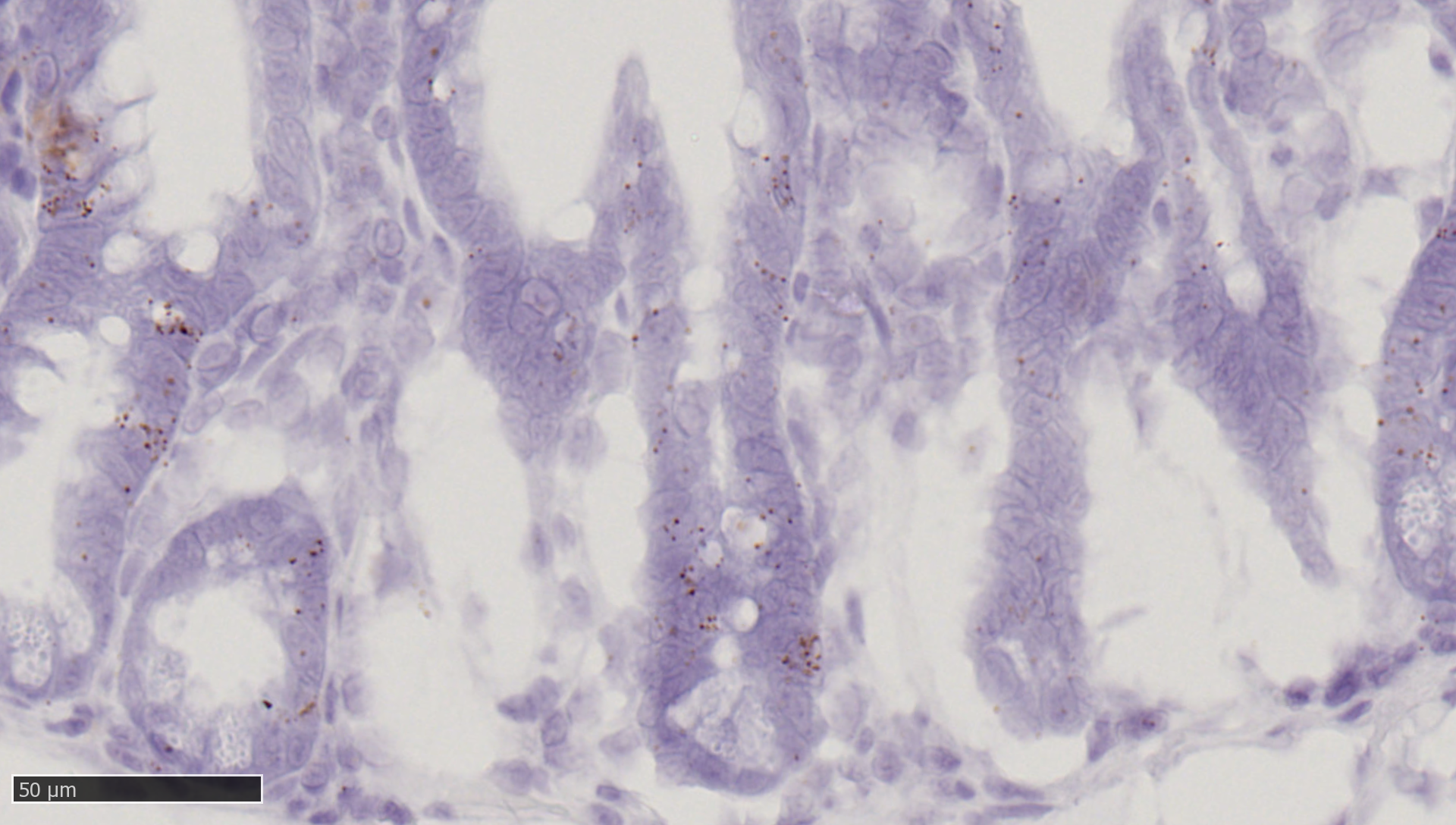

Supplement: Supplementary file 2 — Source Data Fig. 1 [file 44319_2023_13_MOESM2_ESM.zip › Figure 1/1B/1B Ileum Ffar2.tif]

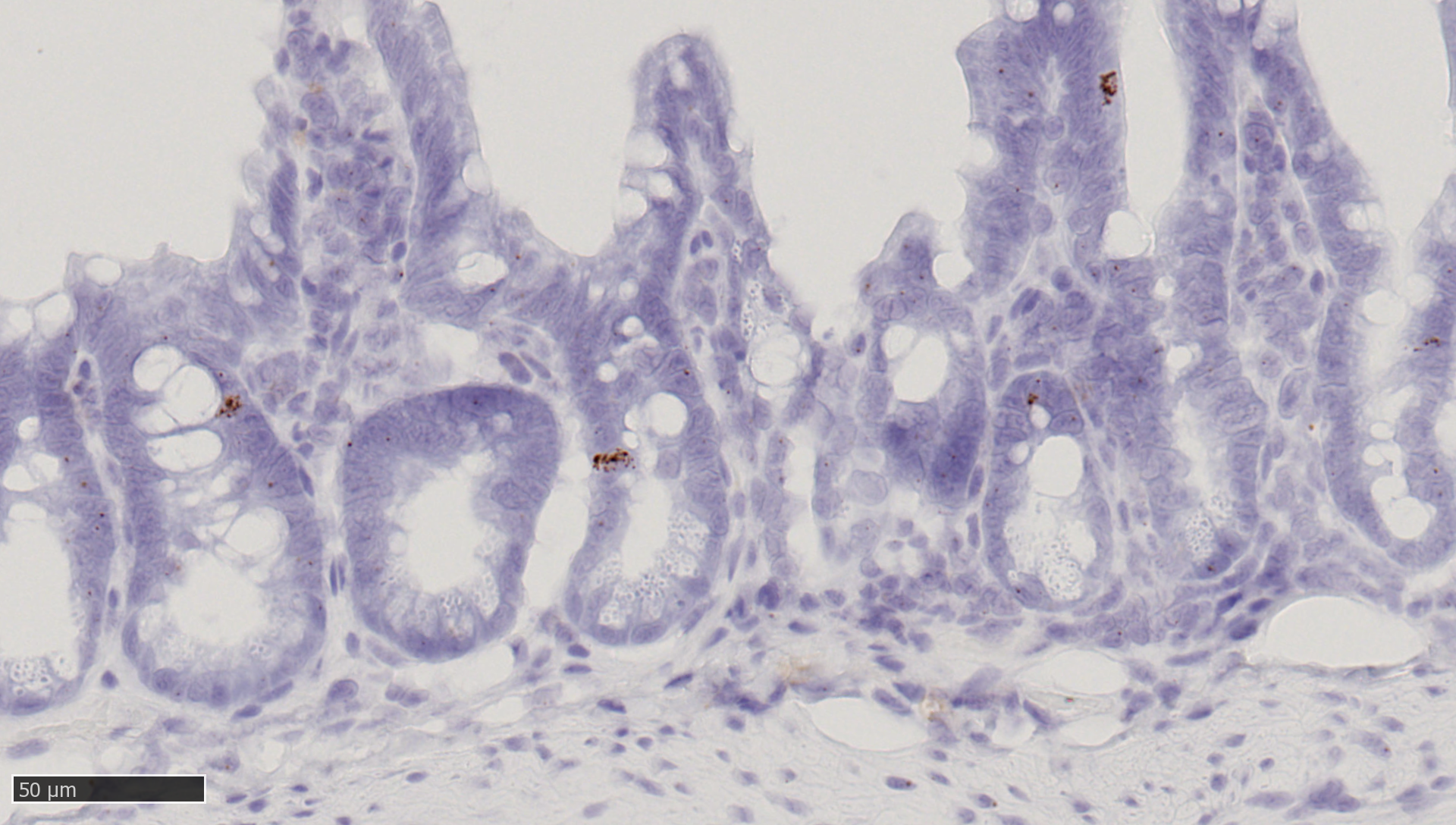

Supplement: Supplementary file 2 — Source Data Fig. 1 [file 44319_2023_13_MOESM2_ESM.zip › Figure 1/1B/1B Ileum Ffar3.tif]

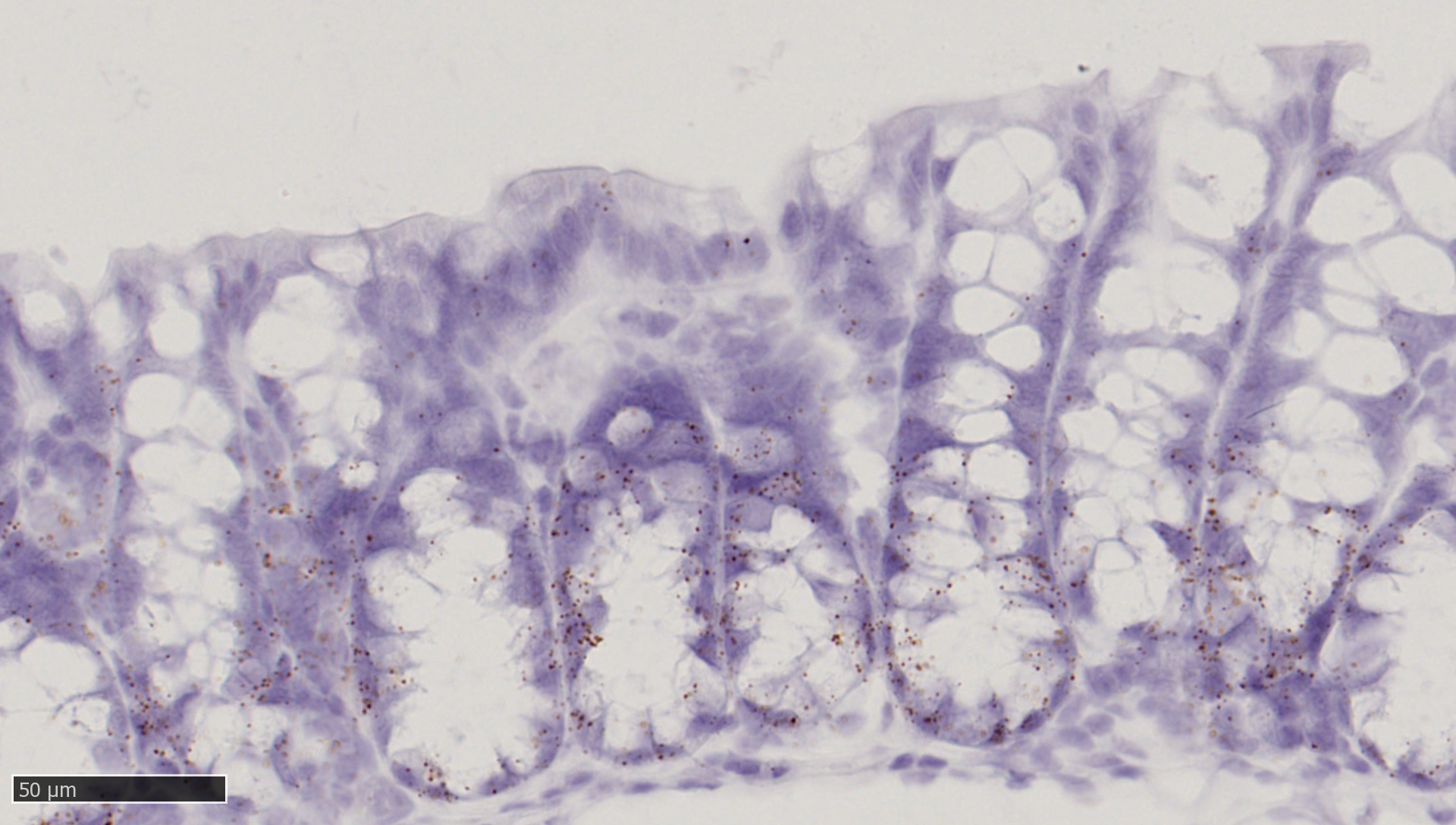

Supplement: Supplementary file 2 — Source Data Fig. 1 [file 44319_2023_13_MOESM2_ESM.zip › Figure 1/1B/1B Proximal Colon Ffar2.tif]

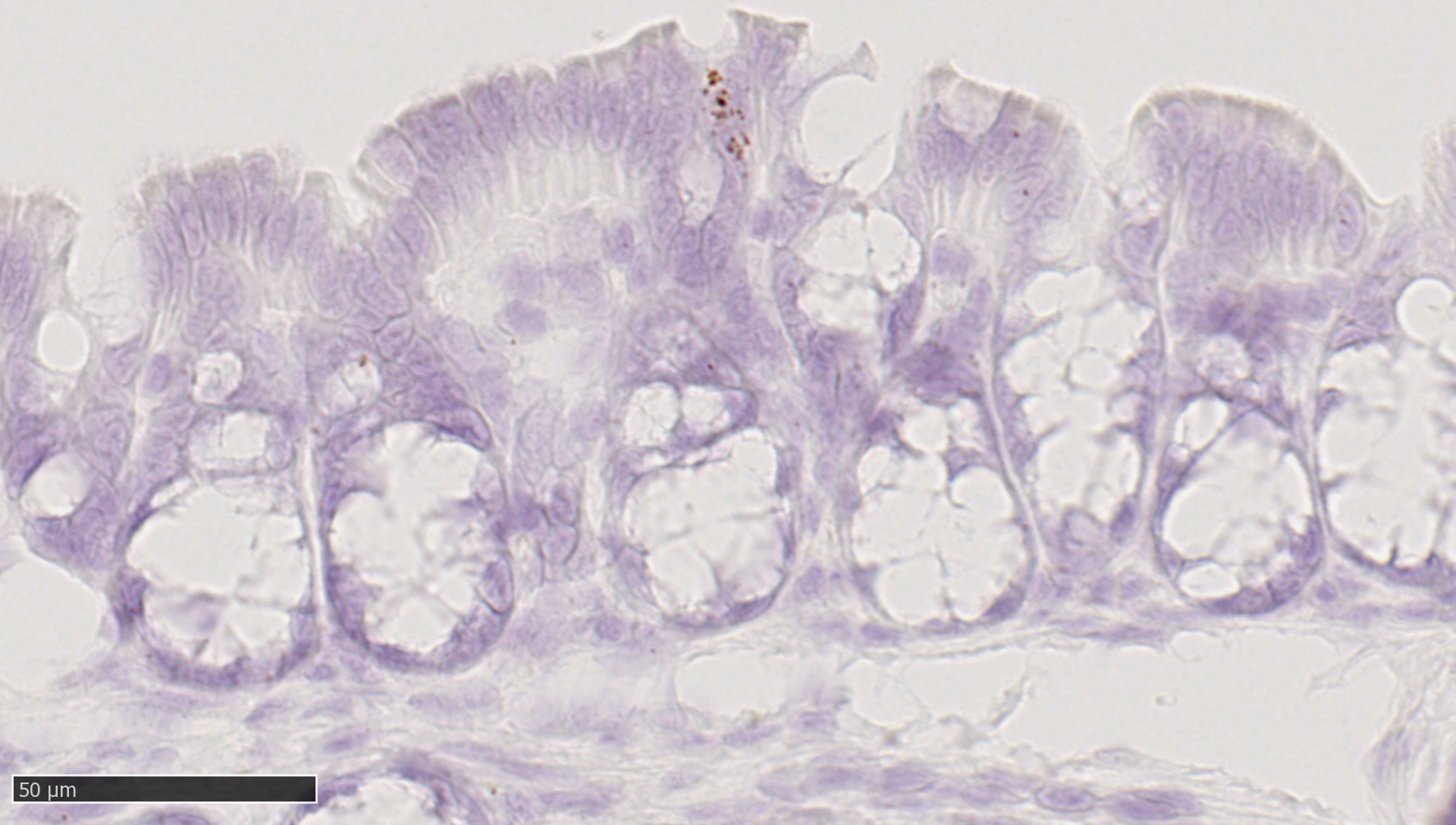

Supplement: Supplementary file 2 — Source Data Fig. 1 [file 44319_2023_13_MOESM2_ESM.zip › Figure 1/1B/1B Proximal Colon Ffar3.tif]

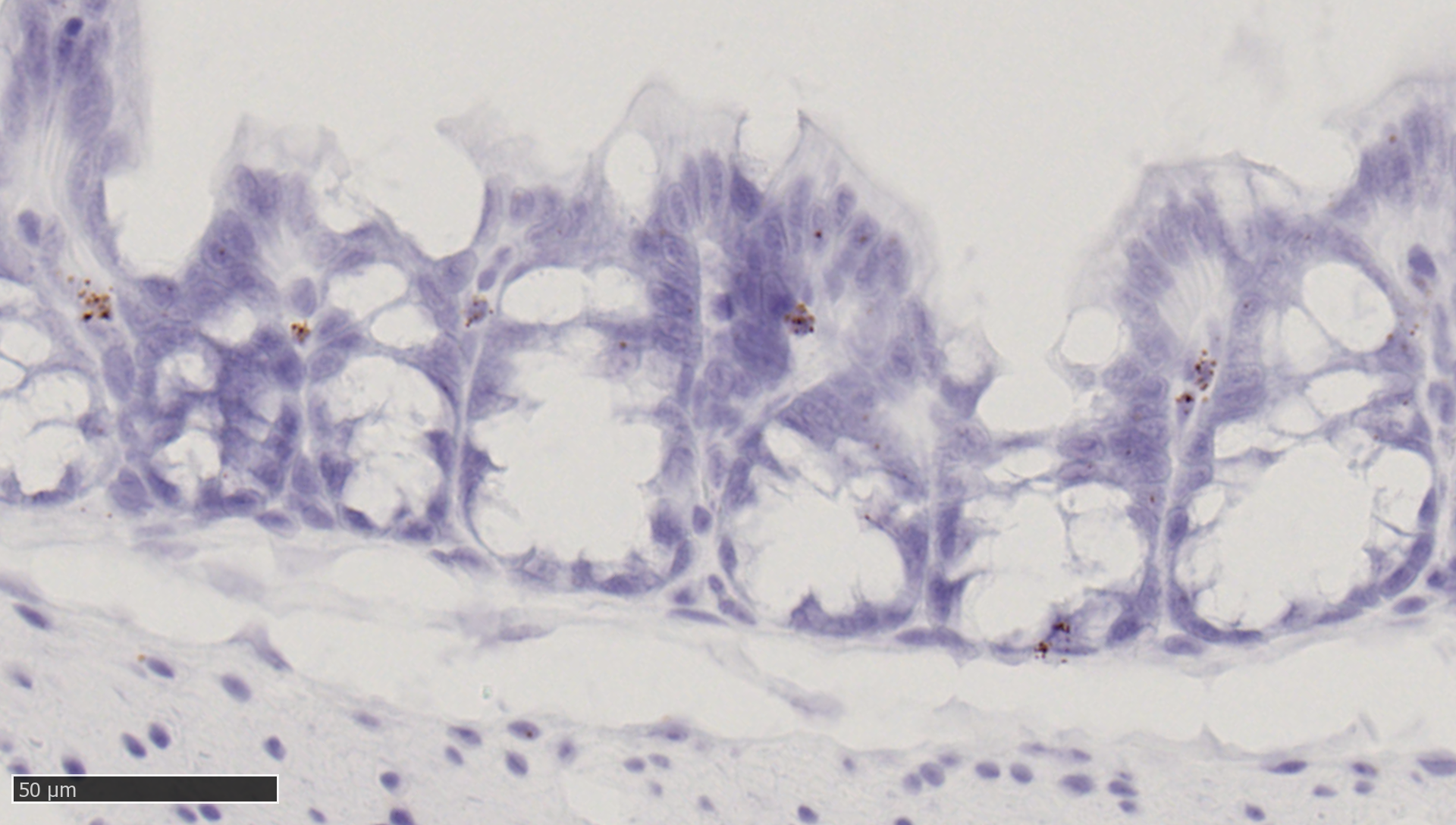

Supplement: Supplementary file 2 — Source Data Fig. 1 [file 44319_2023_13_MOESM2_ESM.zip › Figure 1/1B/1B Proximal Colon Olfr558.tif]

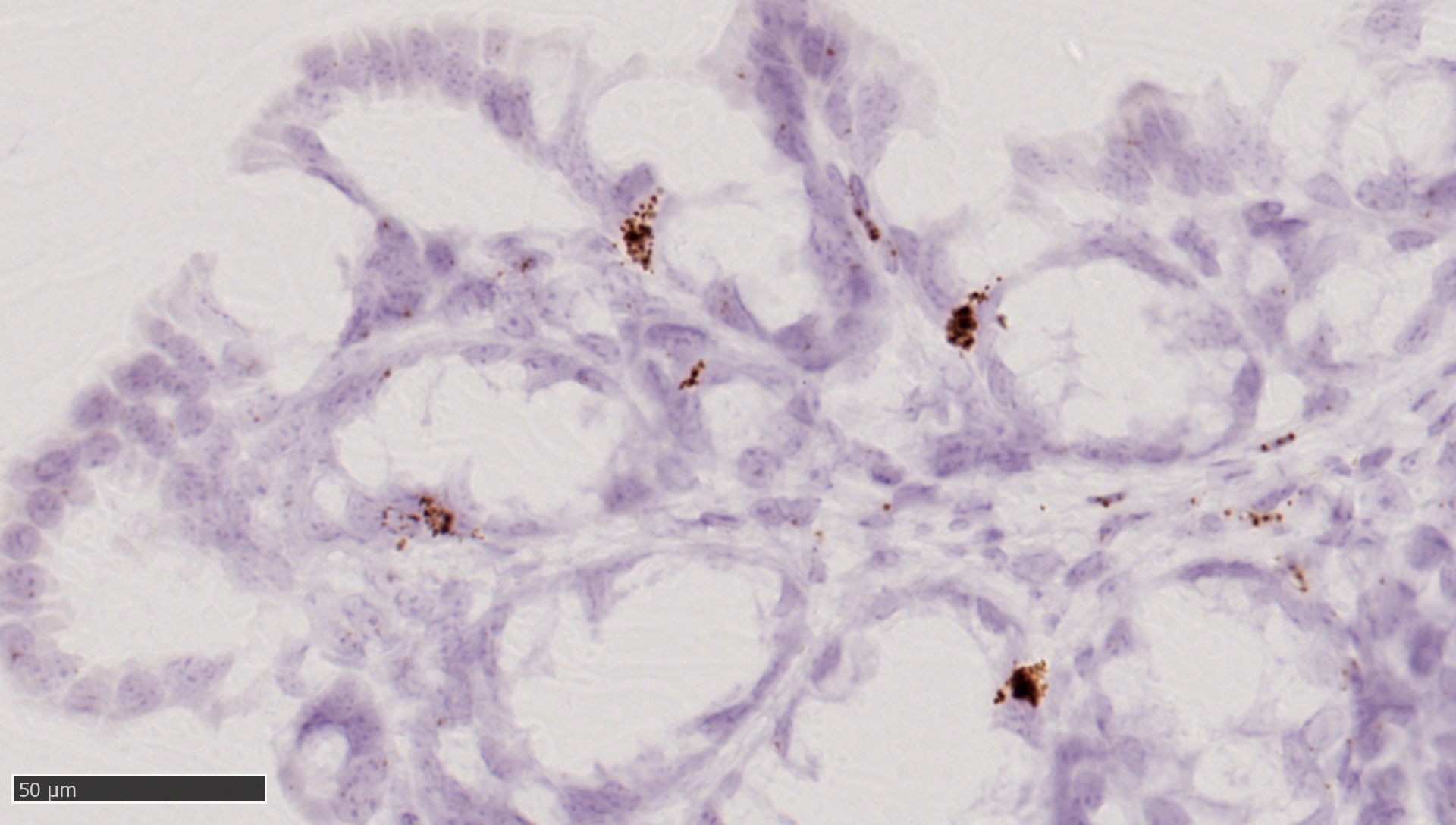

Supplement: Supplementary file 2 — Source Data Fig. 1 [file 44319_2023_13_MOESM2_ESM.zip › Figure 1/1B/1B Proximal Colon Olfr78.tif]

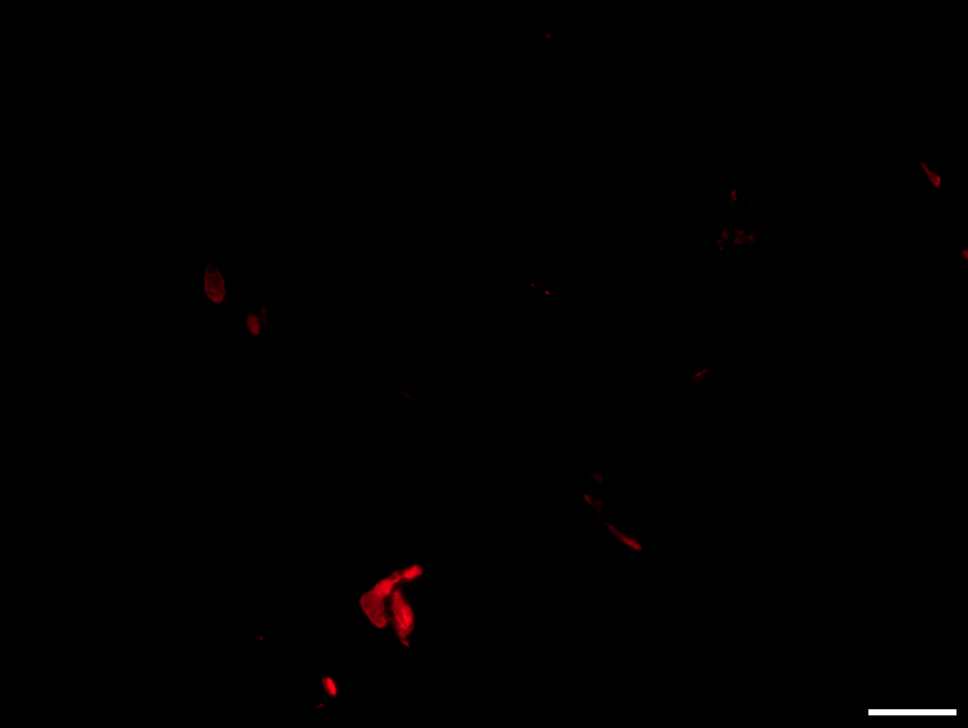

Supplement: Supplementary file 3 — Source Data Fig. 2 [file 44319_2023_13_MOESM3_ESM.zip › Figure 2/2F/5-HT Distal Colon.tif]

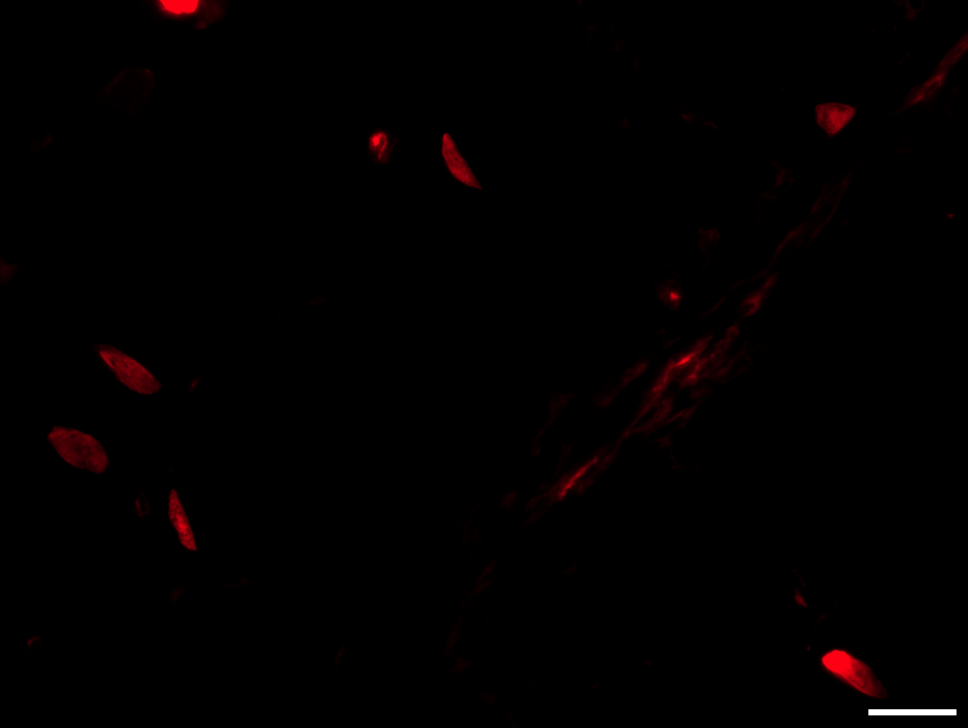

Supplement: Supplementary file 3 — Source Data Fig. 2 [file 44319_2023_13_MOESM3_ESM.zip › Figure 2/2F/5-HT Proximal Colon.tif]

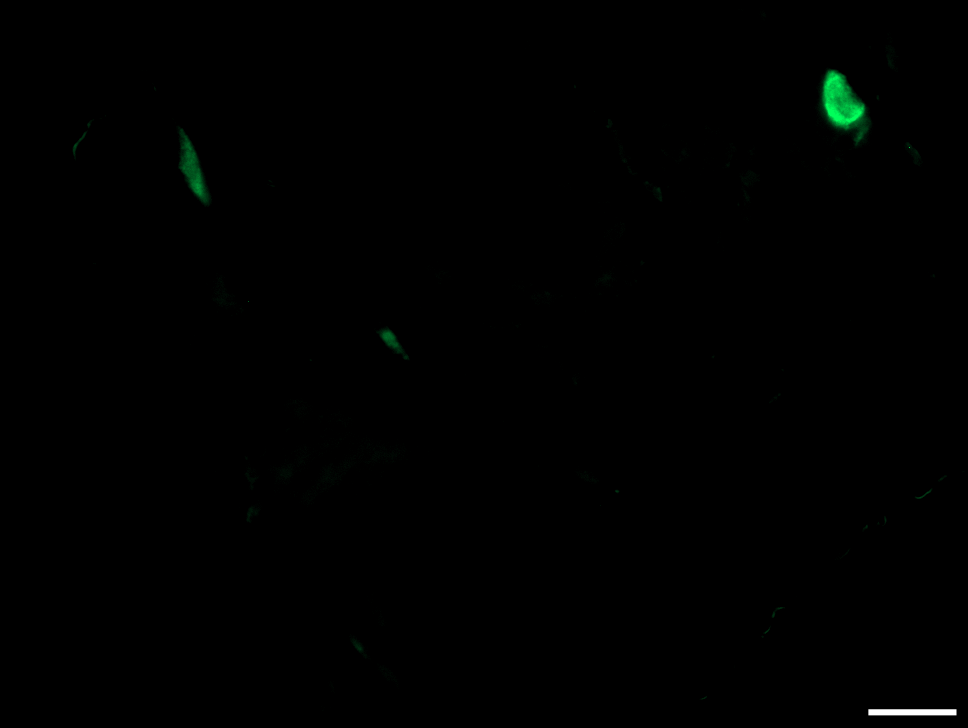

Supplement: Supplementary file 3 — Source Data Fig. 2 [file 44319_2023_13_MOESM3_ESM.zip › Figure 2/2F/GFP without 5-HT Distal Colon.tif]

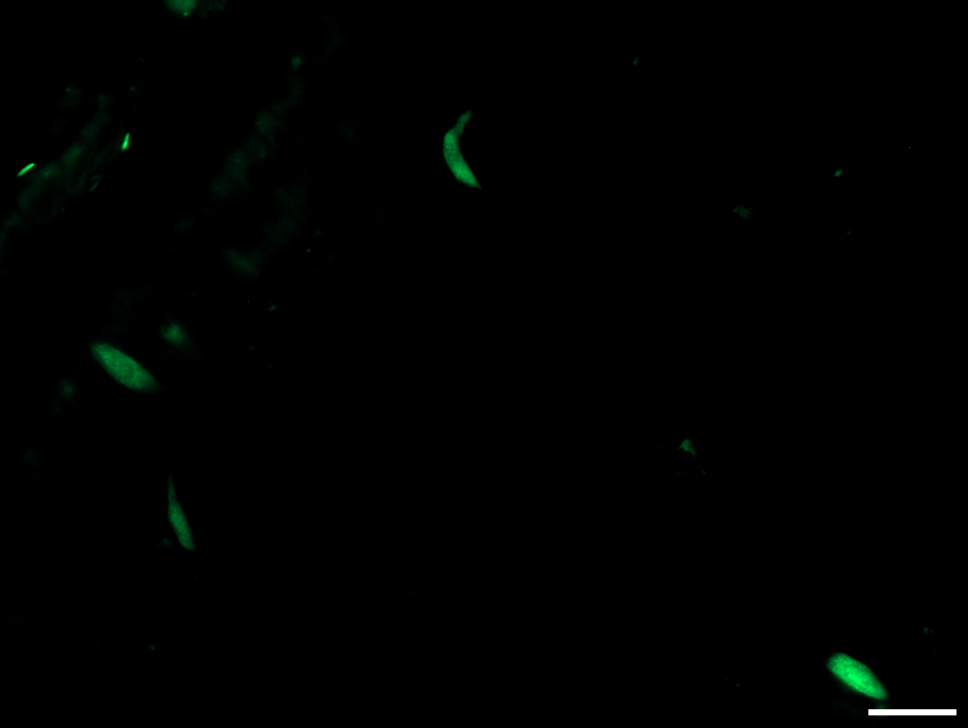

Supplement: Supplementary file 3 — Source Data Fig. 2 [file 44319_2023_13_MOESM3_ESM.zip › Figure 2/2F/GFP without 5-HT Proximal Colon.tif]

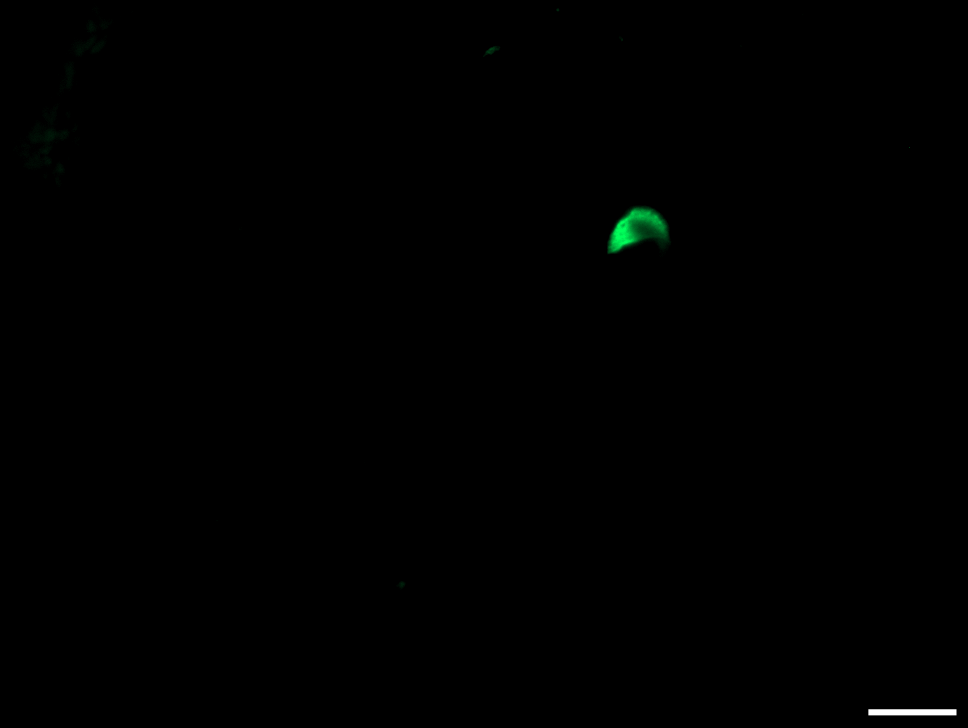

Supplement: Supplementary file 3 — Source Data Fig. 2 [file 44319_2023_13_MOESM3_ESM.zip › Figure 2/2F/GFP withtout PYY Distal Colon.tif]

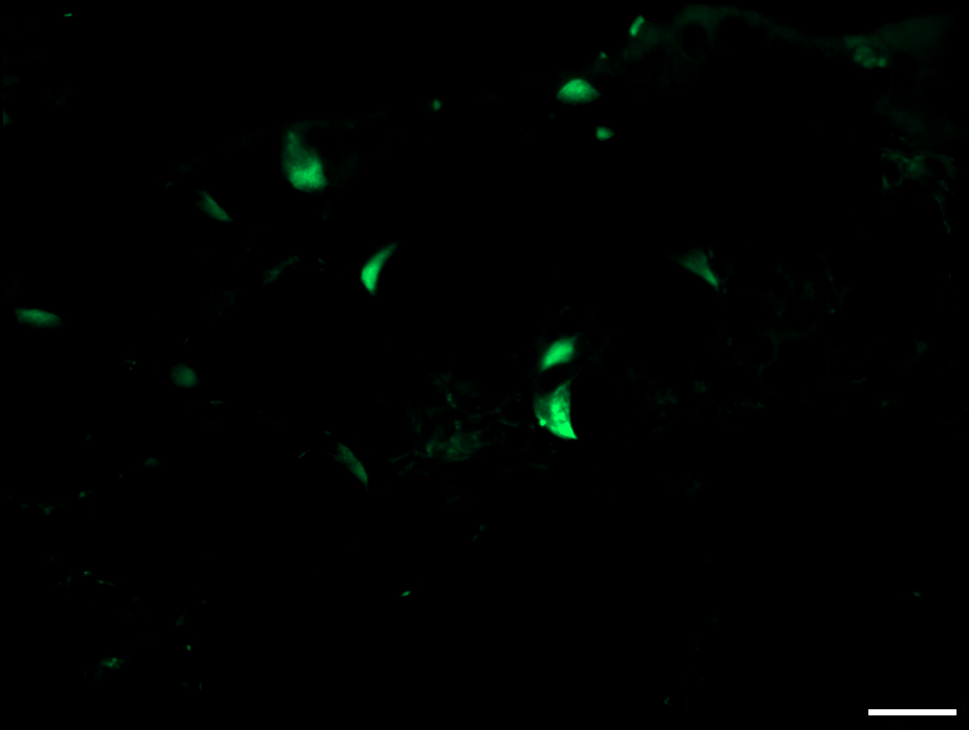

Supplement: Supplementary file 3 — Source Data Fig. 2 [file 44319_2023_13_MOESM3_ESM.zip › Figure 2/2F/GFP withtout PYY Proximal Colon.tif]

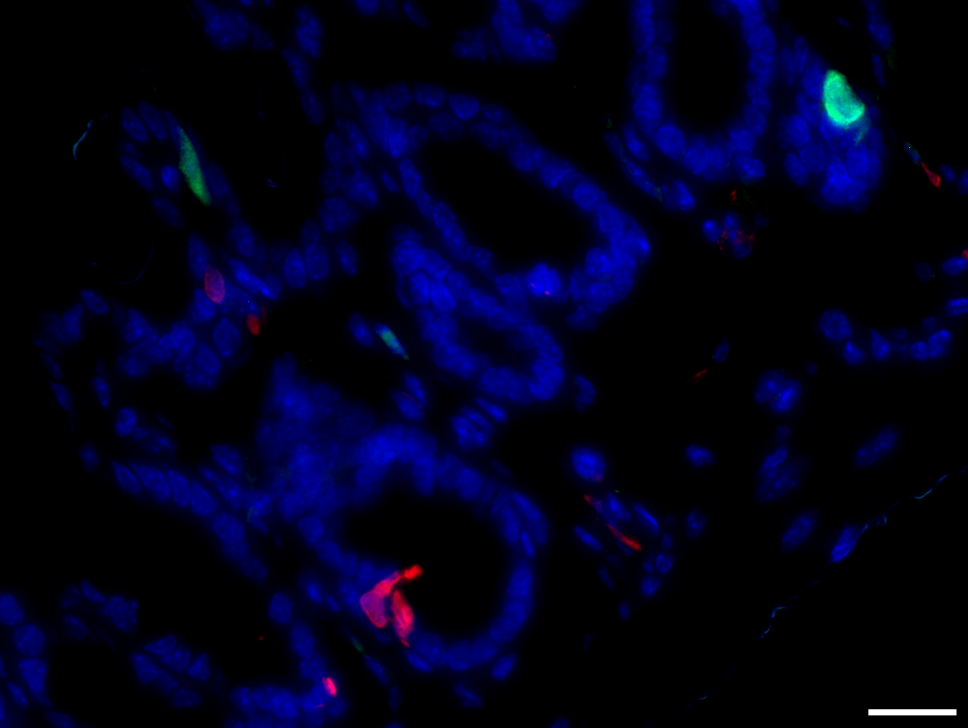

Supplement: Supplementary file 3 — Source Data Fig. 2 [file 44319_2023_13_MOESM3_ESM.zip › Figure 2/2F/Merge 5-HT GFP Distal Colon.tif]

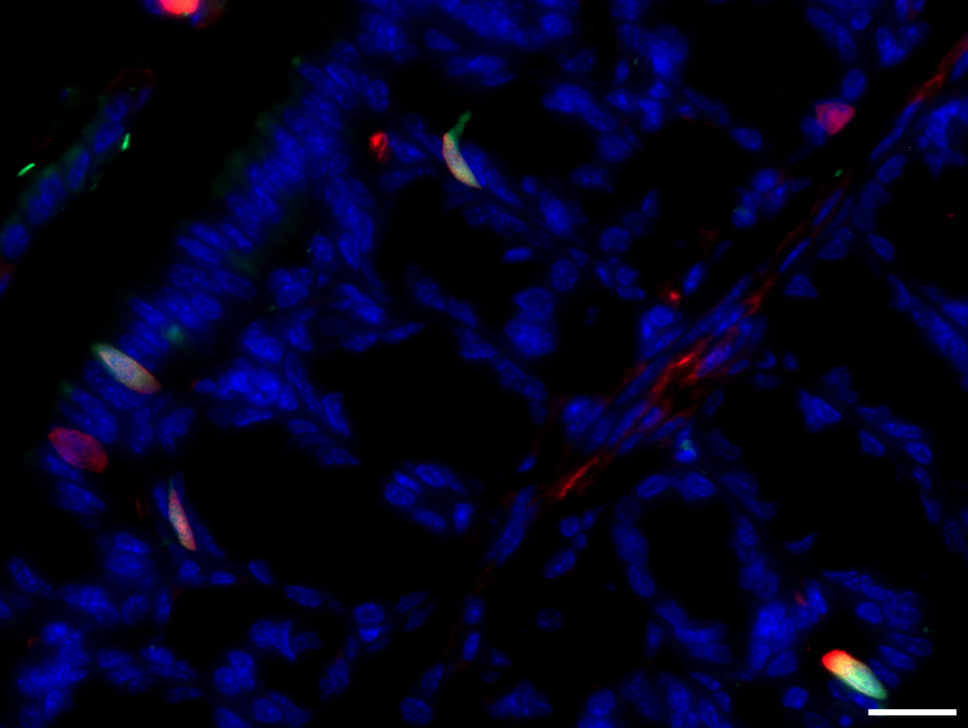

Supplement: Supplementary file 3 — Source Data Fig. 2 [file 44319_2023_13_MOESM3_ESM.zip › Figure 2/2F/Merge 5-HT GFP Proximal Colon.tif]

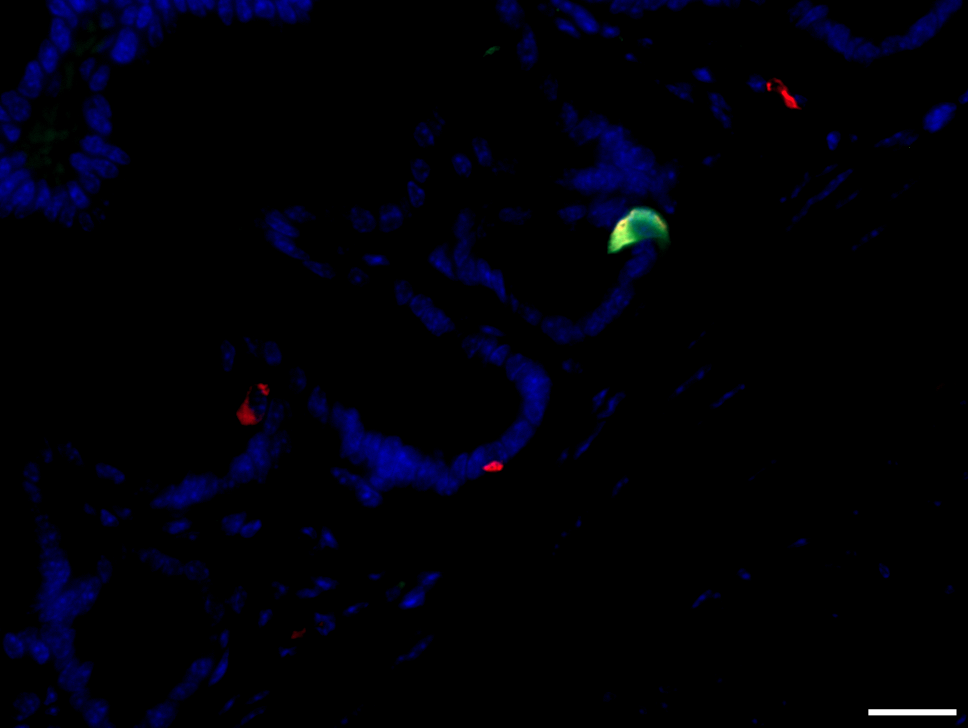

Supplement: Supplementary file 3 — Source Data Fig. 2 [file 44319_2023_13_MOESM3_ESM.zip › Figure 2/2F/Merge PYY GFP Distal Colon.tif]

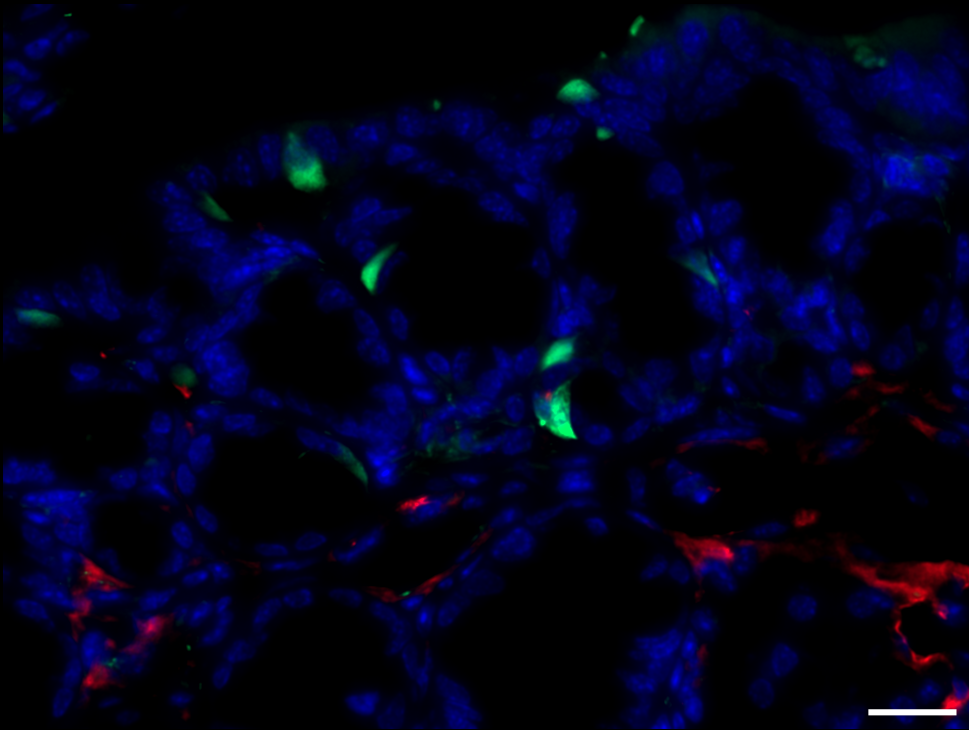

Supplement: Supplementary file 3 — Source Data Fig. 2 [file 44319_2023_13_MOESM3_ESM.zip › Figure 2/2F/Merge PYY GFP Proximal Colon.tif]

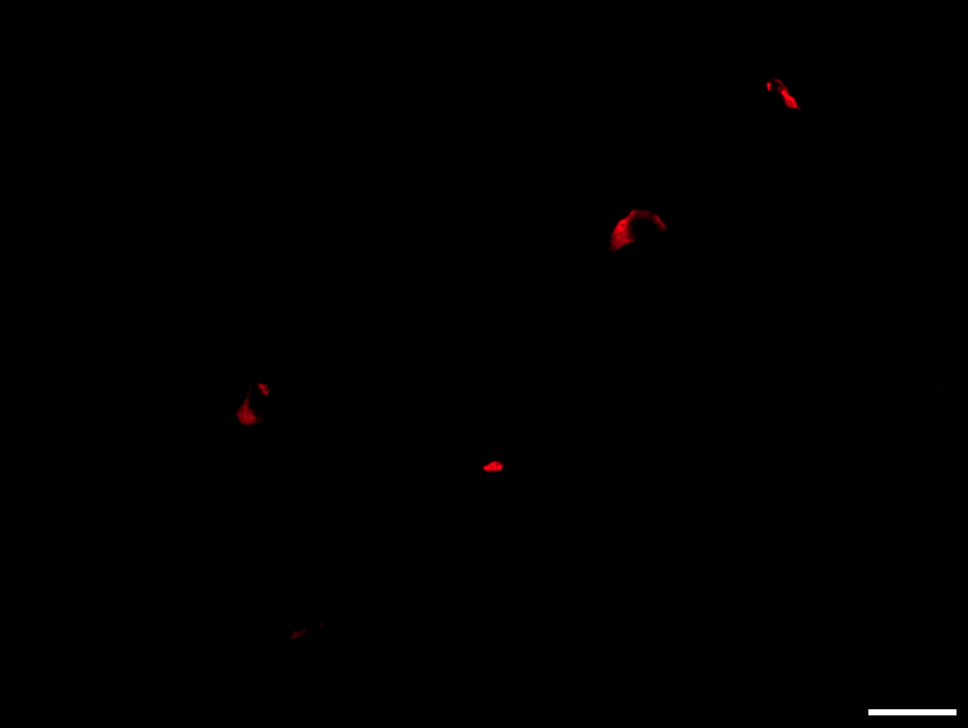

Supplement: Supplementary file 3 — Source Data Fig. 2 [file 44319_2023_13_MOESM3_ESM.zip › Figure 2/2F/PYY Distal Colon.tif]

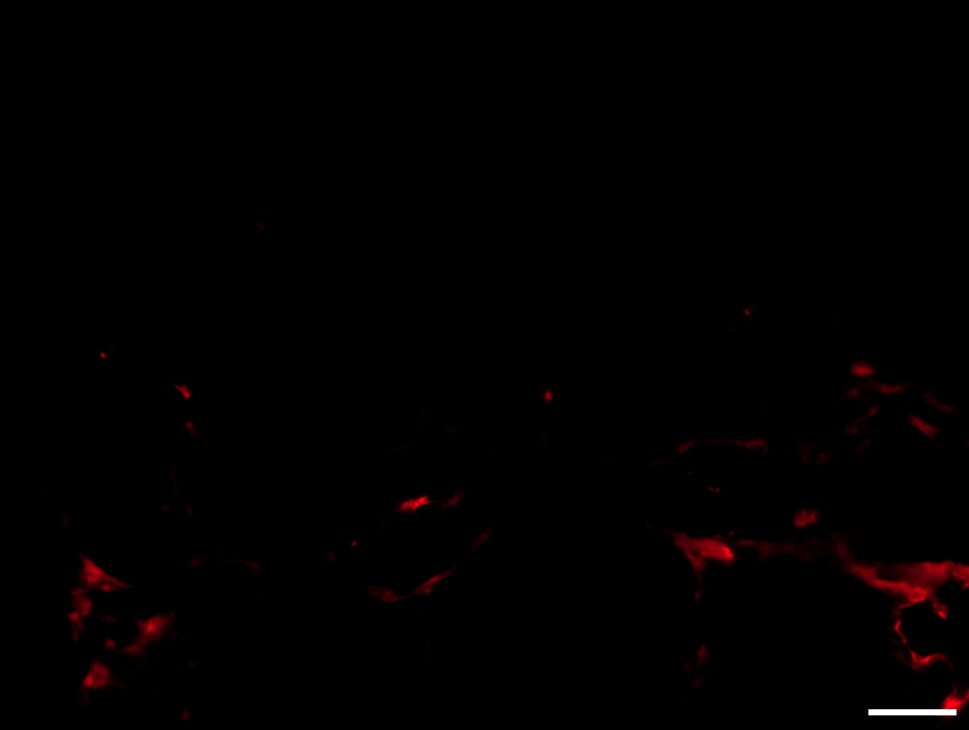

Supplement: Supplementary file 3 — Source Data Fig. 2 [file 44319_2023_13_MOESM3_ESM.zip › Figure 2/2F/PYY Proximal Colon.tif]

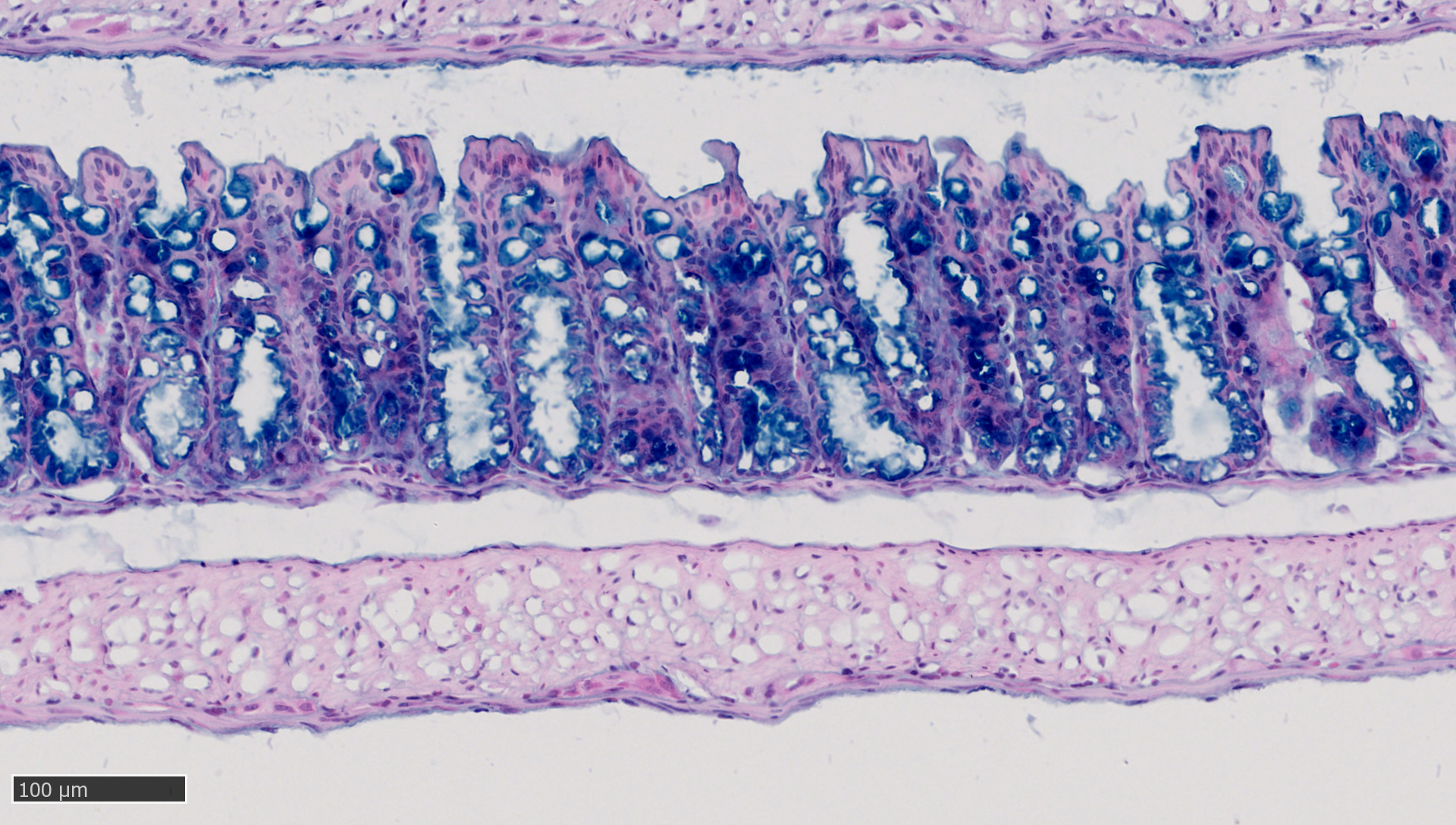

Supplement: Supplementary file 4 — Source Data Fig. 3 [file 44319_2023_13_MOESM4_ESM.zip › Figure 3/3B/Olfr78 KO Alcian Blue Distal Colon.tif]

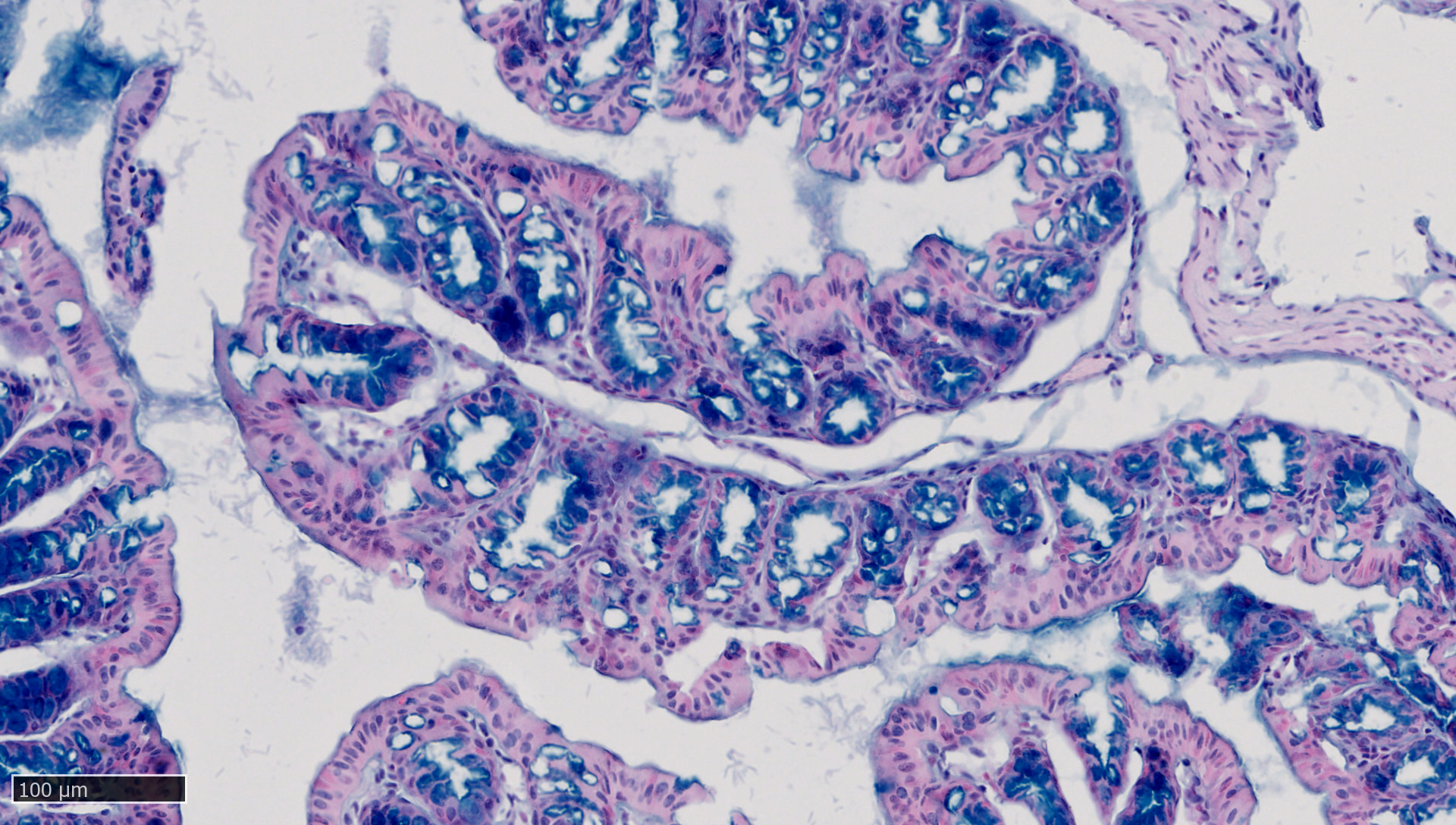

Supplement: Supplementary file 4 — Source Data Fig. 3 [file 44319_2023_13_MOESM4_ESM.zip › Figure 3/3B/Olfr78 KO Alcian Blue Proximal Colon.tif]

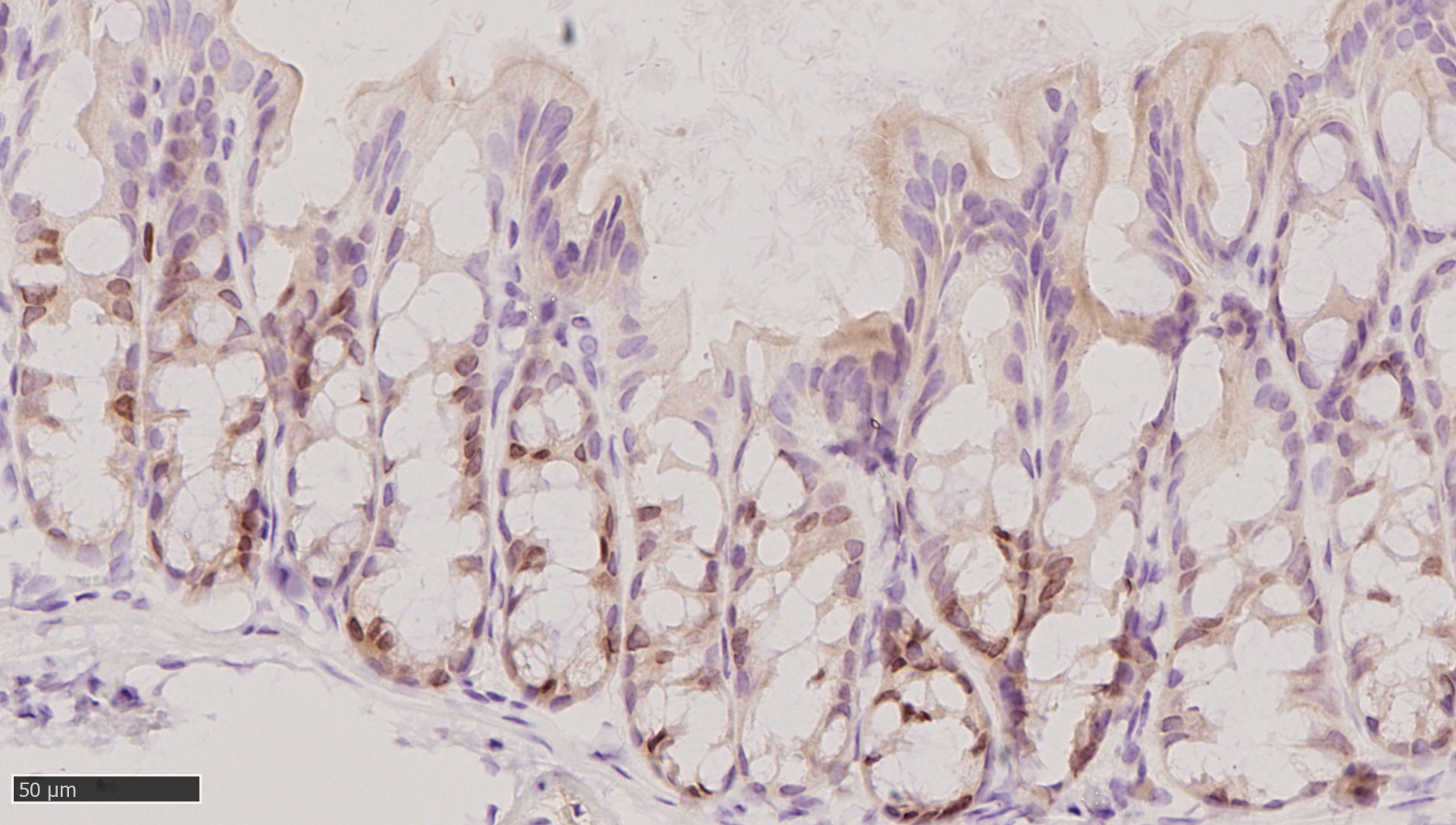

Supplement: Supplementary file 4 — Source Data Fig. 3 [file 44319_2023_13_MOESM4_ESM.zip › Figure 3/3B/Olfr78 KO KI67 Distal Colon.tif]

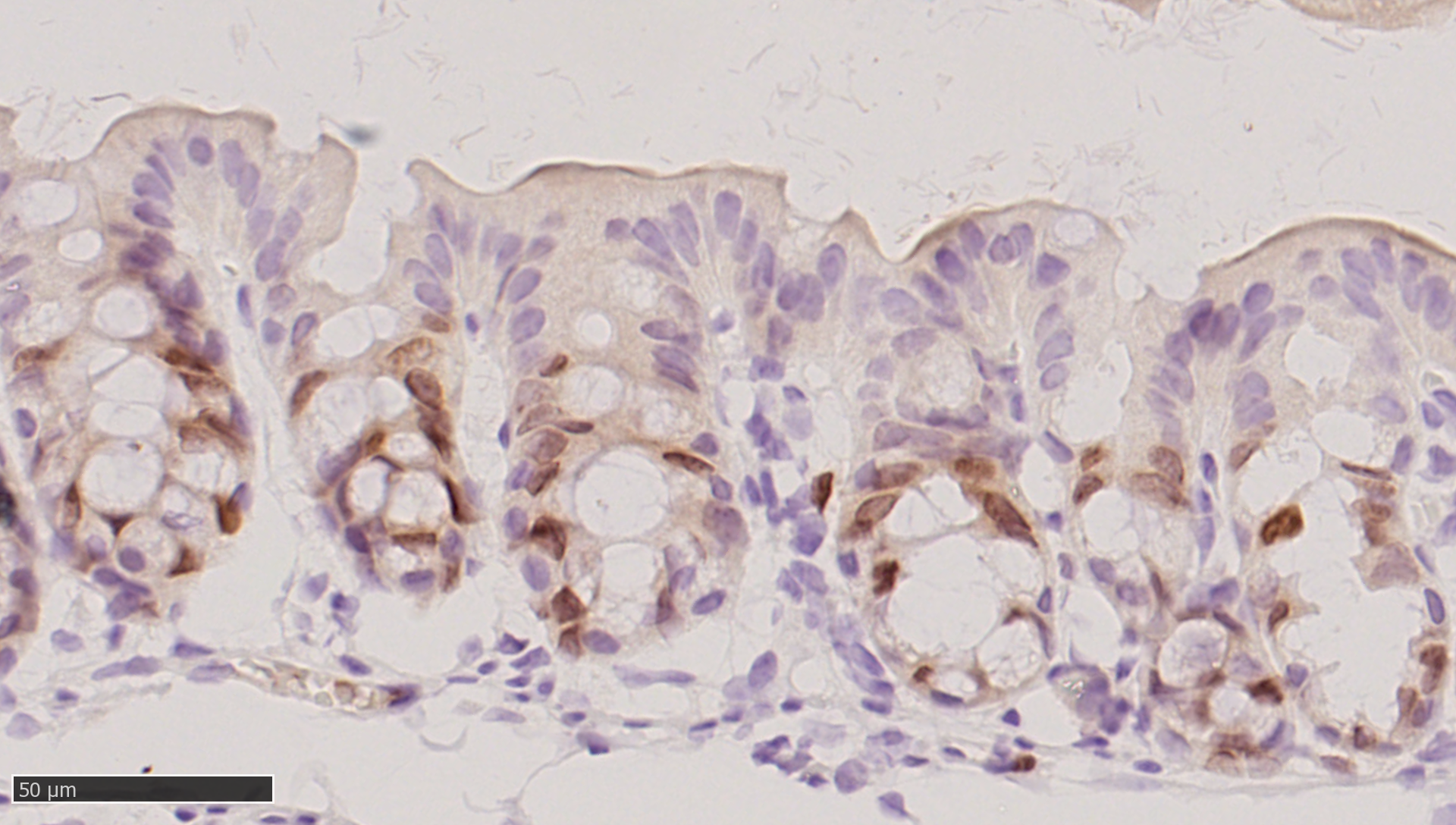

Supplement: Supplementary file 4 — Source Data Fig. 3 [file 44319_2023_13_MOESM4_ESM.zip › Figure 3/3B/Olfr78 KO KI67 Proximal Colon.tif]

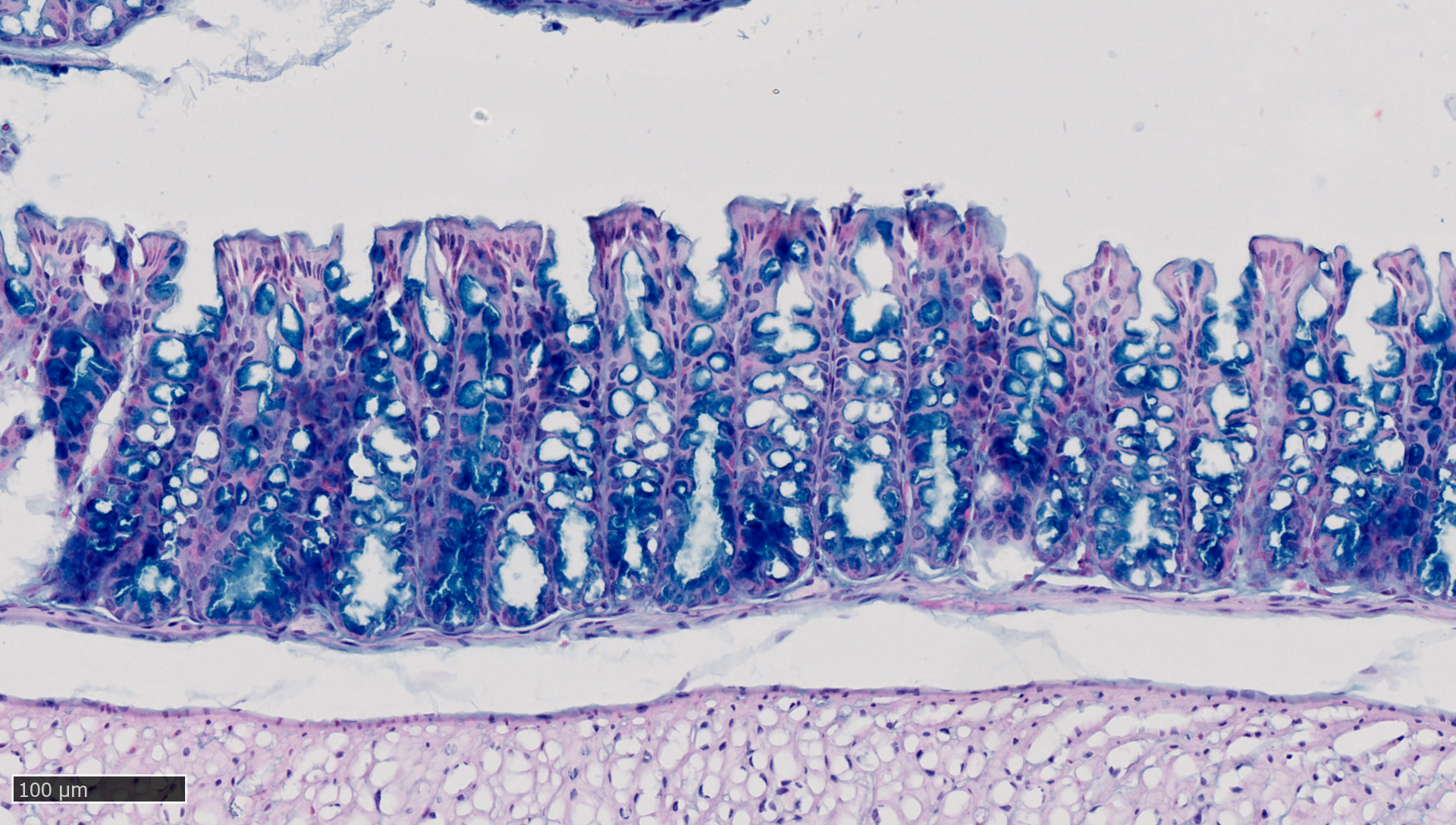

Supplement: Supplementary file 4 — Source Data Fig. 3 [file 44319_2023_13_MOESM4_ESM.zip › Figure 3/3B/Olfr78 WT Alcian Blue Distal Colon.tif]

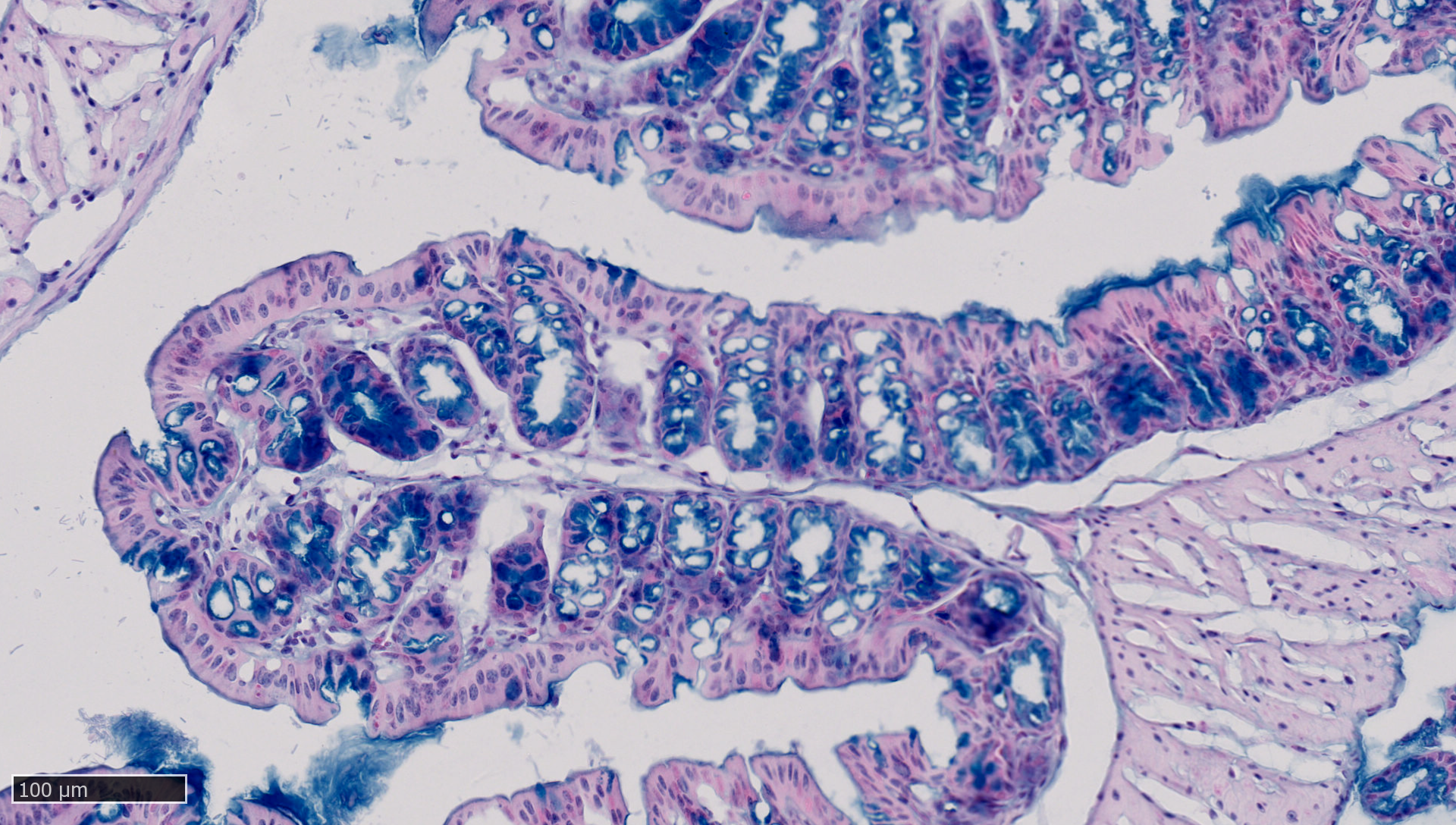

Supplement: Supplementary file 4 — Source Data Fig. 3 [file 44319_2023_13_MOESM4_ESM.zip › Figure 3/3B/Olfr78 WT Alcian Blue Proximal Colon.tif]

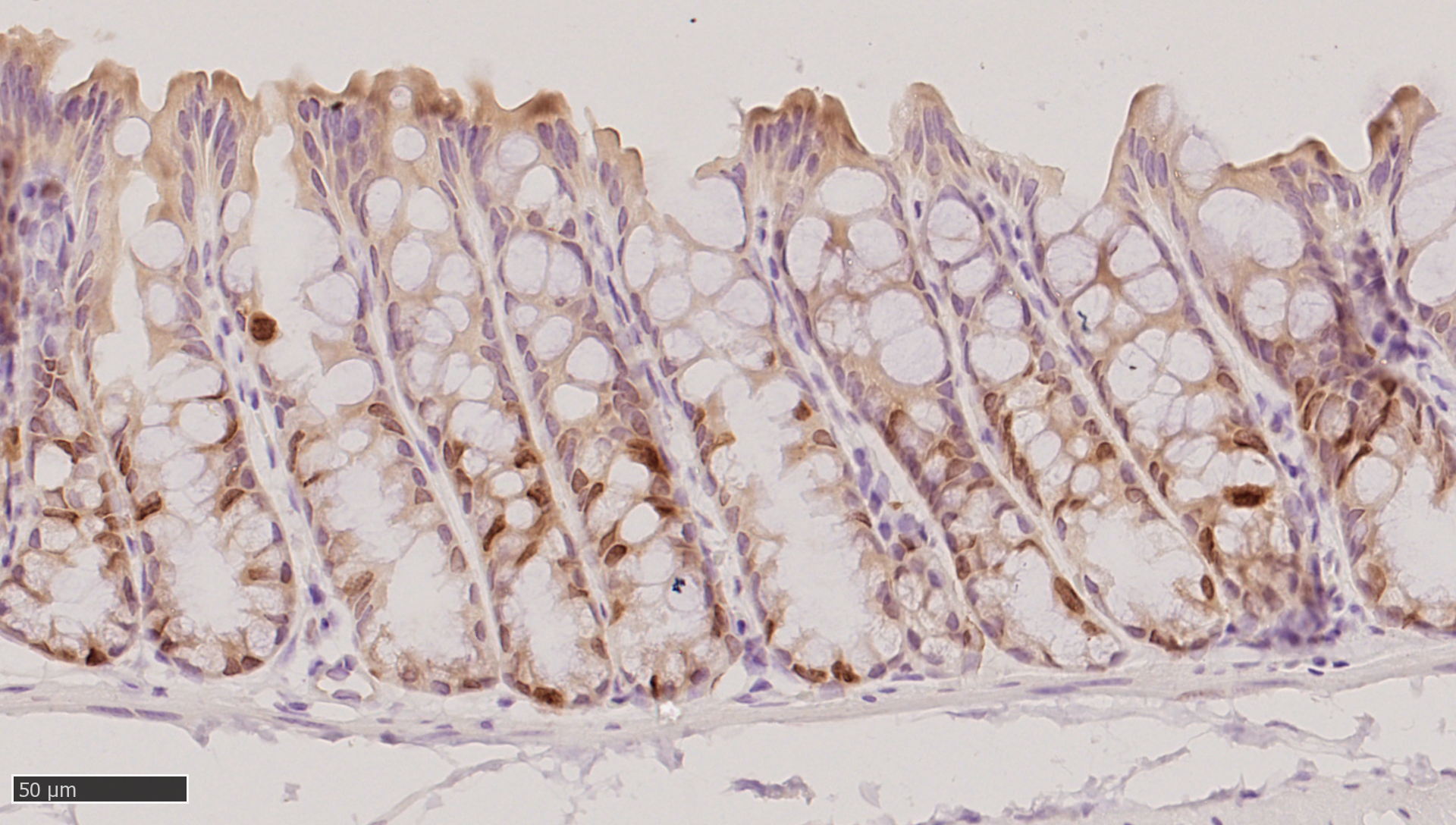

Supplement: Supplementary file 4 — Source Data Fig. 3 [file 44319_2023_13_MOESM4_ESM.zip › Figure 3/3B/Olfr78 WT KI67 Distal Colon.tif]

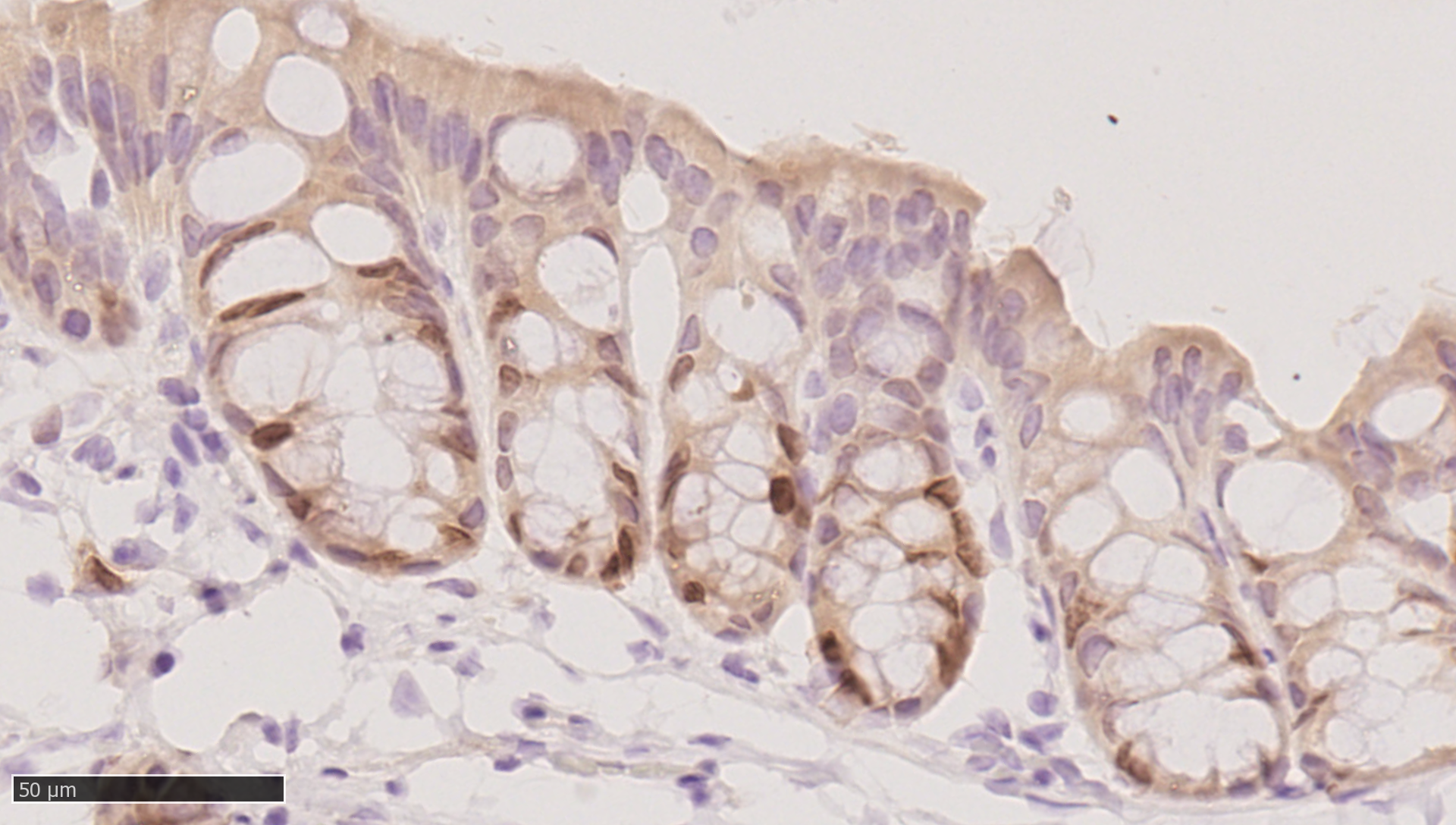

Supplement: Supplementary file 4 — Source Data Fig. 3 [file 44319_2023_13_MOESM4_ESM.zip › Figure 3/3B/Olfr78 WT KI67 Proximal Colon.tif]

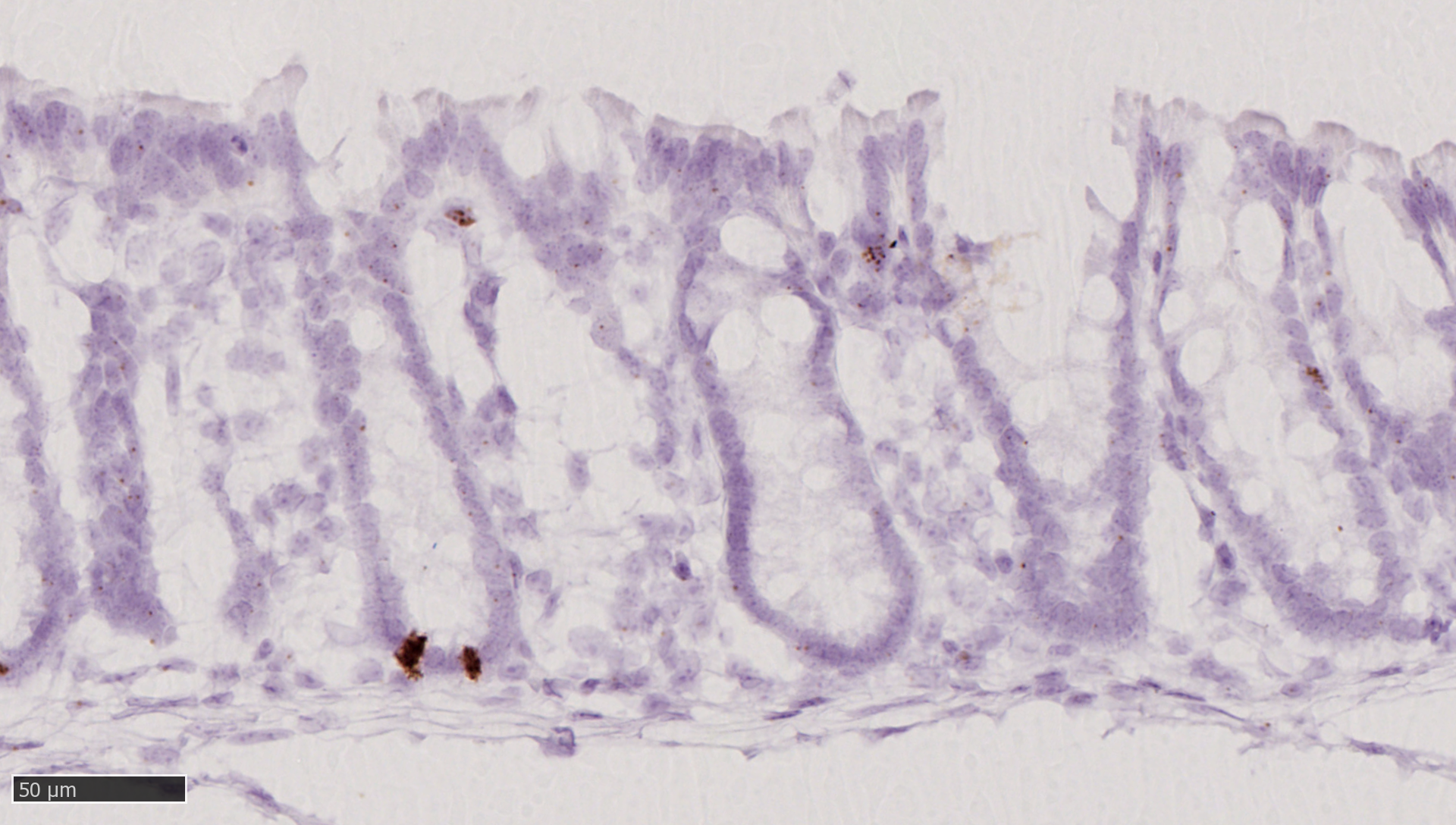

Supplement: Supplementary file 5 — Source Data Fig. 4 [file 44319_2023_13_MOESM5_ESM.zip › Figure 4/4B/low view col dist Vil1Cre+ 78++.tif]

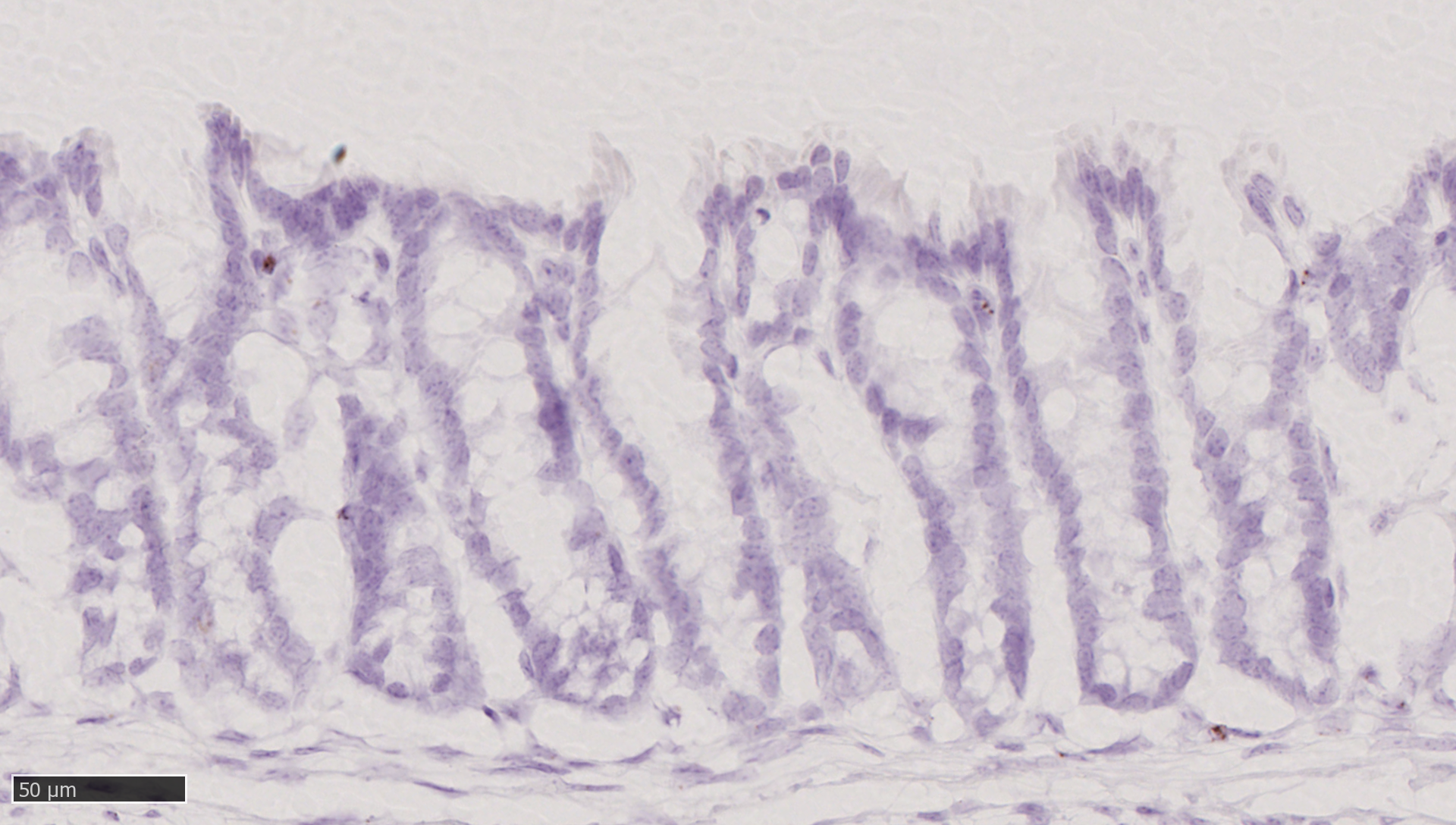

Supplement: Supplementary file 5 — Source Data Fig. 4 [file 44319_2023_13_MOESM5_ESM.zip › Figure 4/4B/low view col dist Vil1Cre+ 78FxFx.tif]

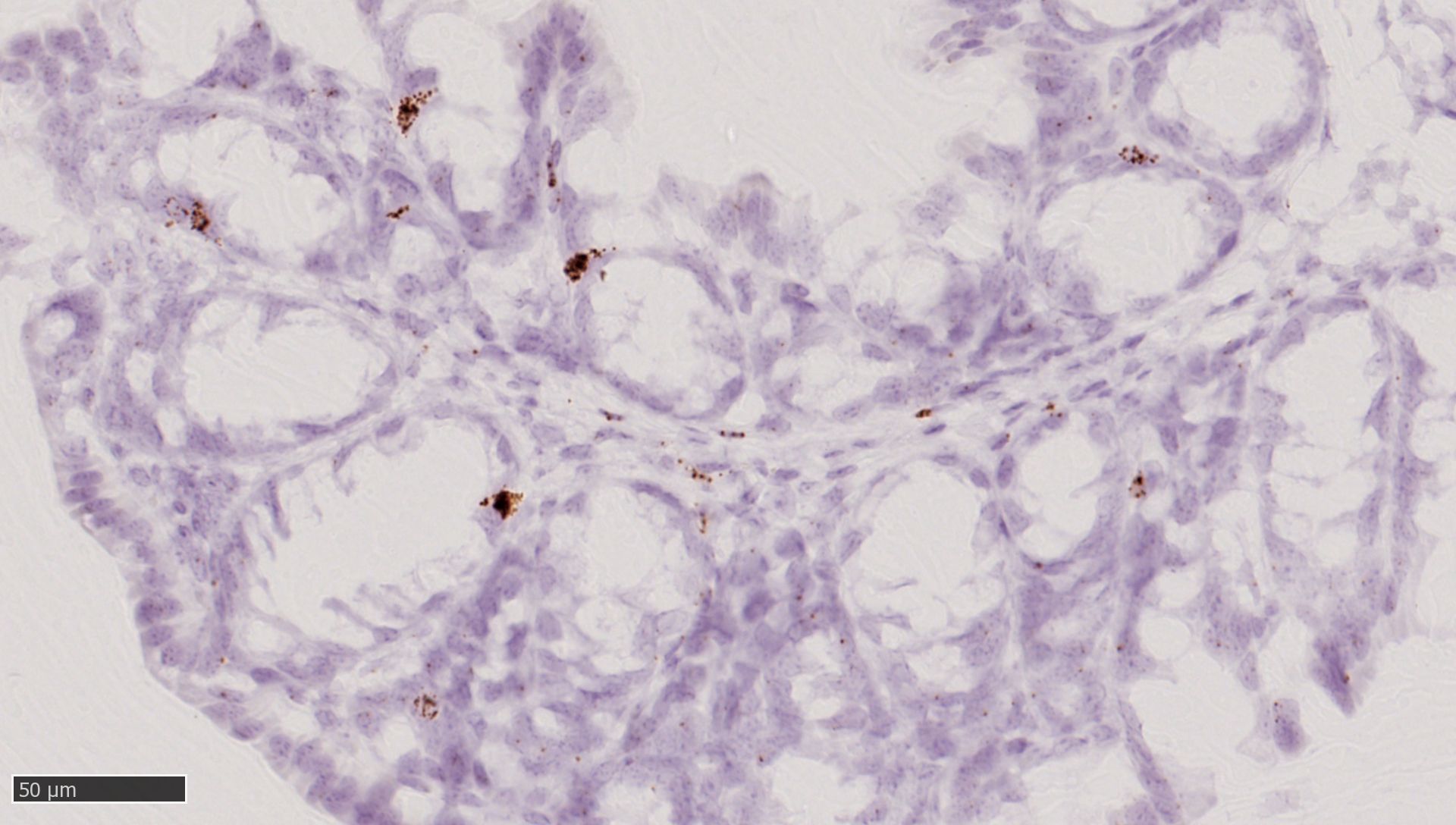

Supplement: Supplementary file 5 — Source Data Fig. 4 [file 44319_2023_13_MOESM5_ESM.zip › Figure 4/4B/low view col prox Vil1Cre+ 78++.tif]

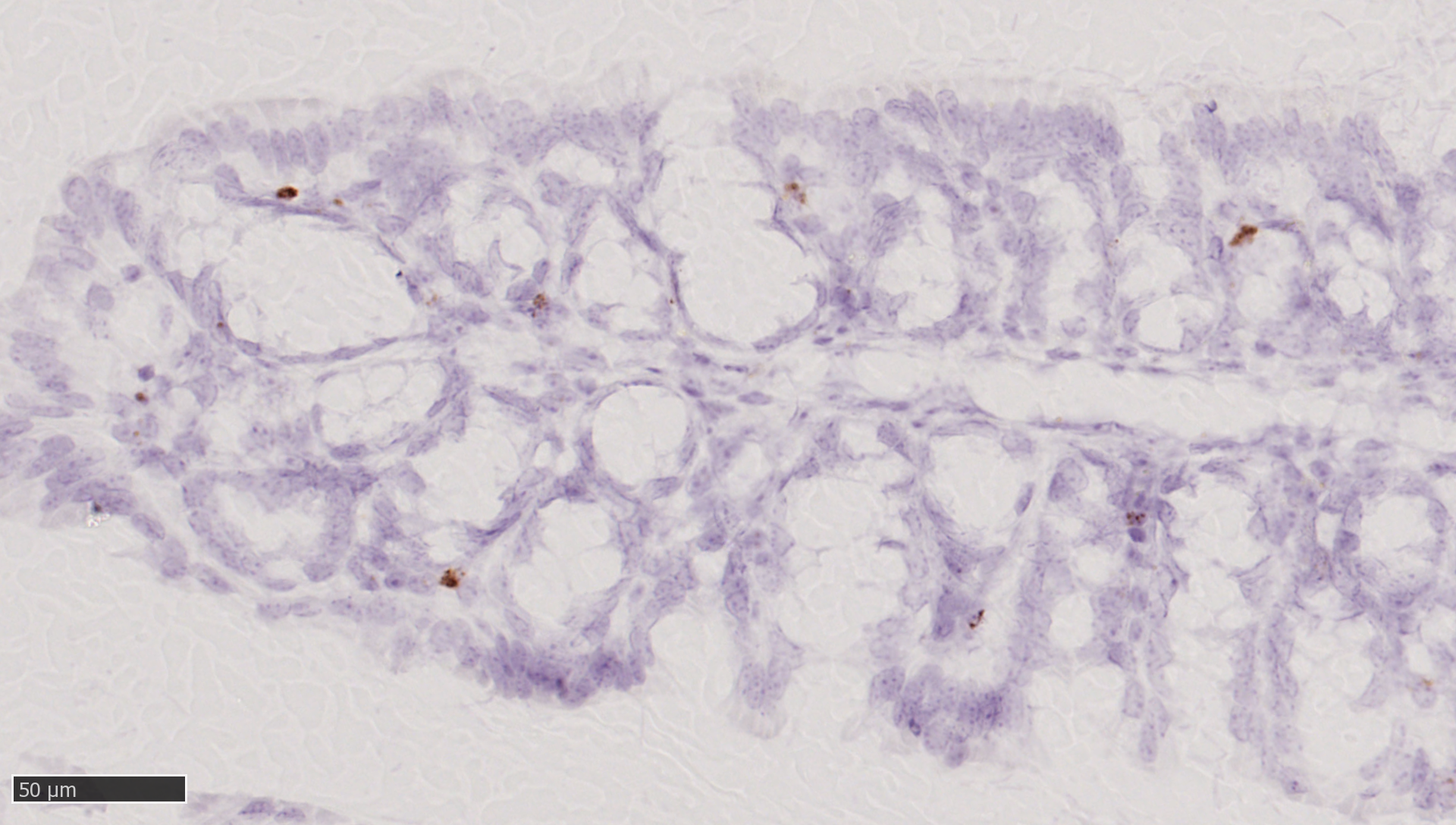

Supplement: Supplementary file 5 — Source Data Fig. 4 [file 44319_2023_13_MOESM5_ESM.zip › Figure 4/4B/low view colprox Vil1Cre+ 78FxFx.tif]

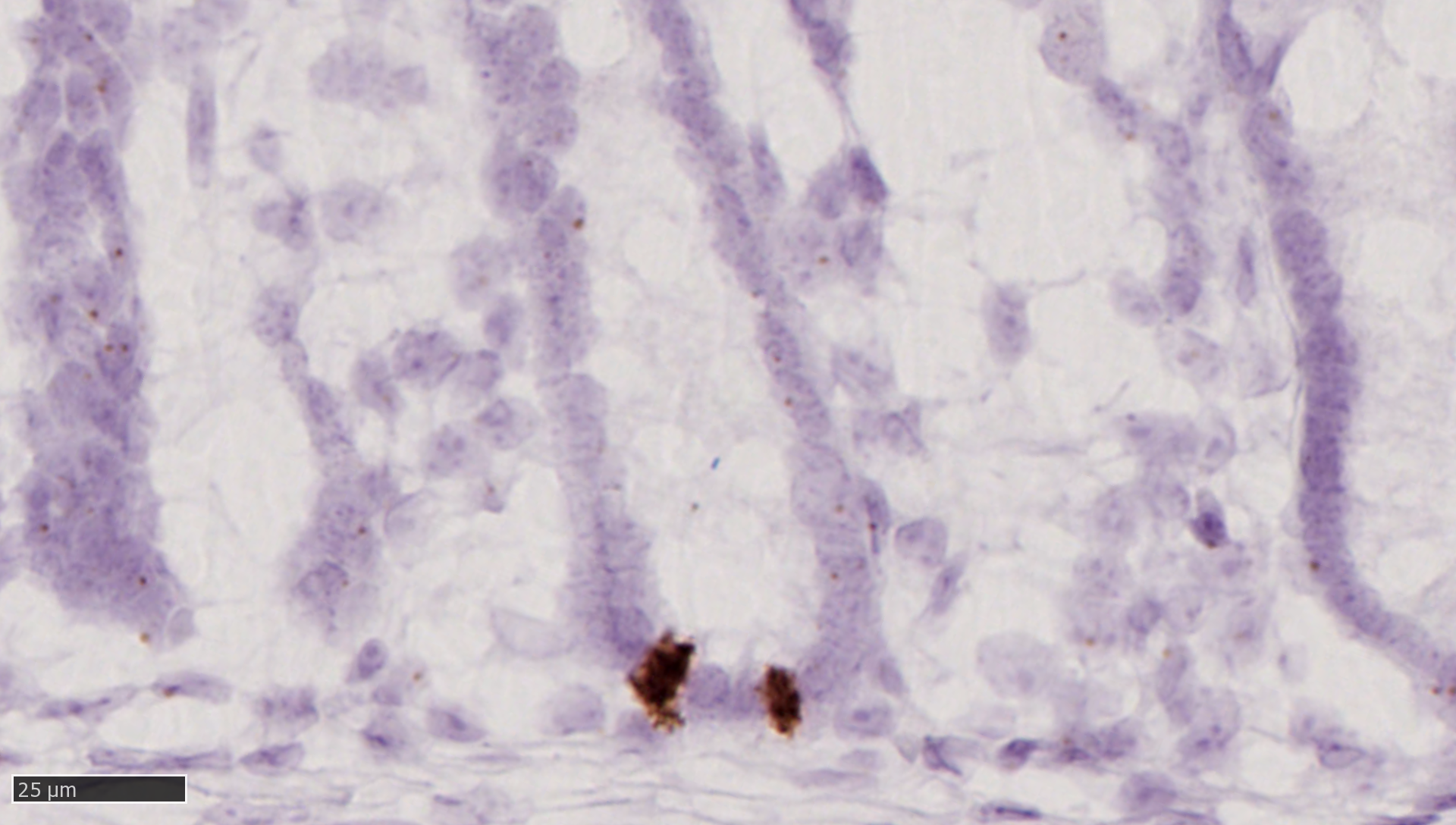

Supplement: Supplementary file 5 — Source Data Fig. 4 [file 44319_2023_13_MOESM5_ESM.zip › Figure 4/4B/zoom col dist Vil1Cre+ 78++.tif]

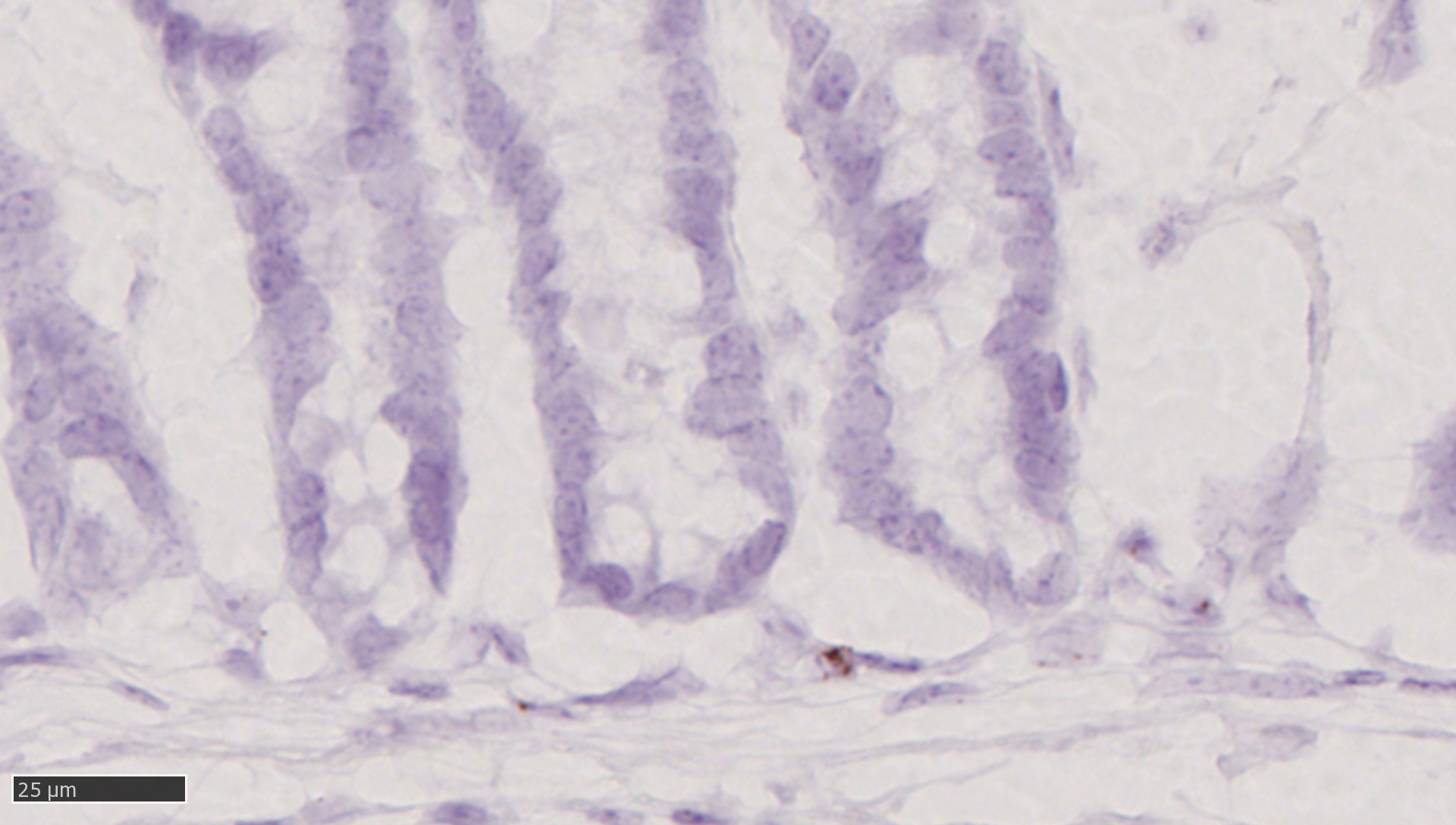

Supplement: Supplementary file 5 — Source Data Fig. 4 [file 44319_2023_13_MOESM5_ESM.zip › Figure 4/4B/zoom col dist Vil1Cre+ 78FxFx.tif]

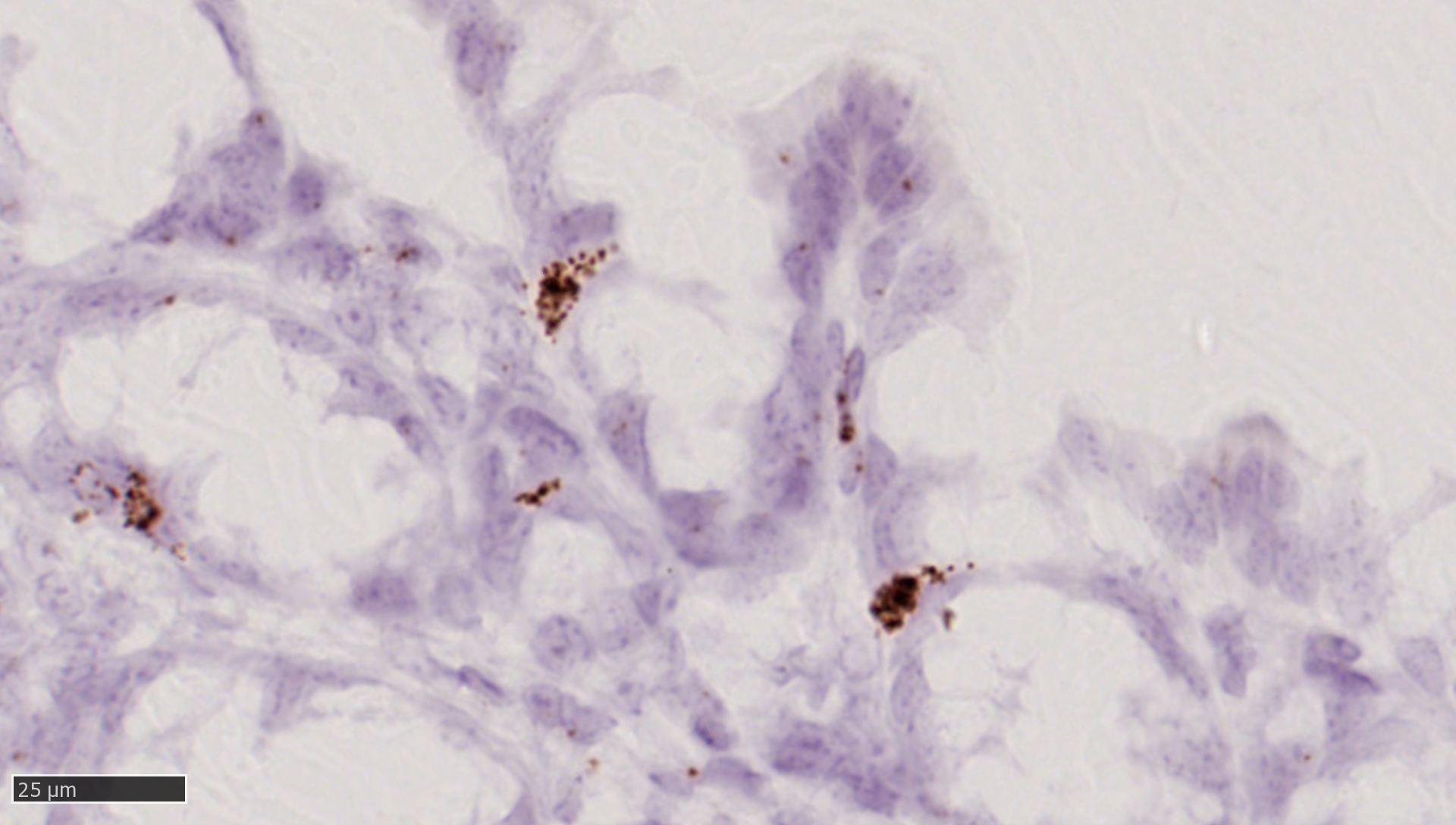

Supplement: Supplementary file 5 — Source Data Fig. 4 [file 44319_2023_13_MOESM5_ESM.zip › Figure 4/4B/zoom col prox Vil1Cre+ 78++.tif]

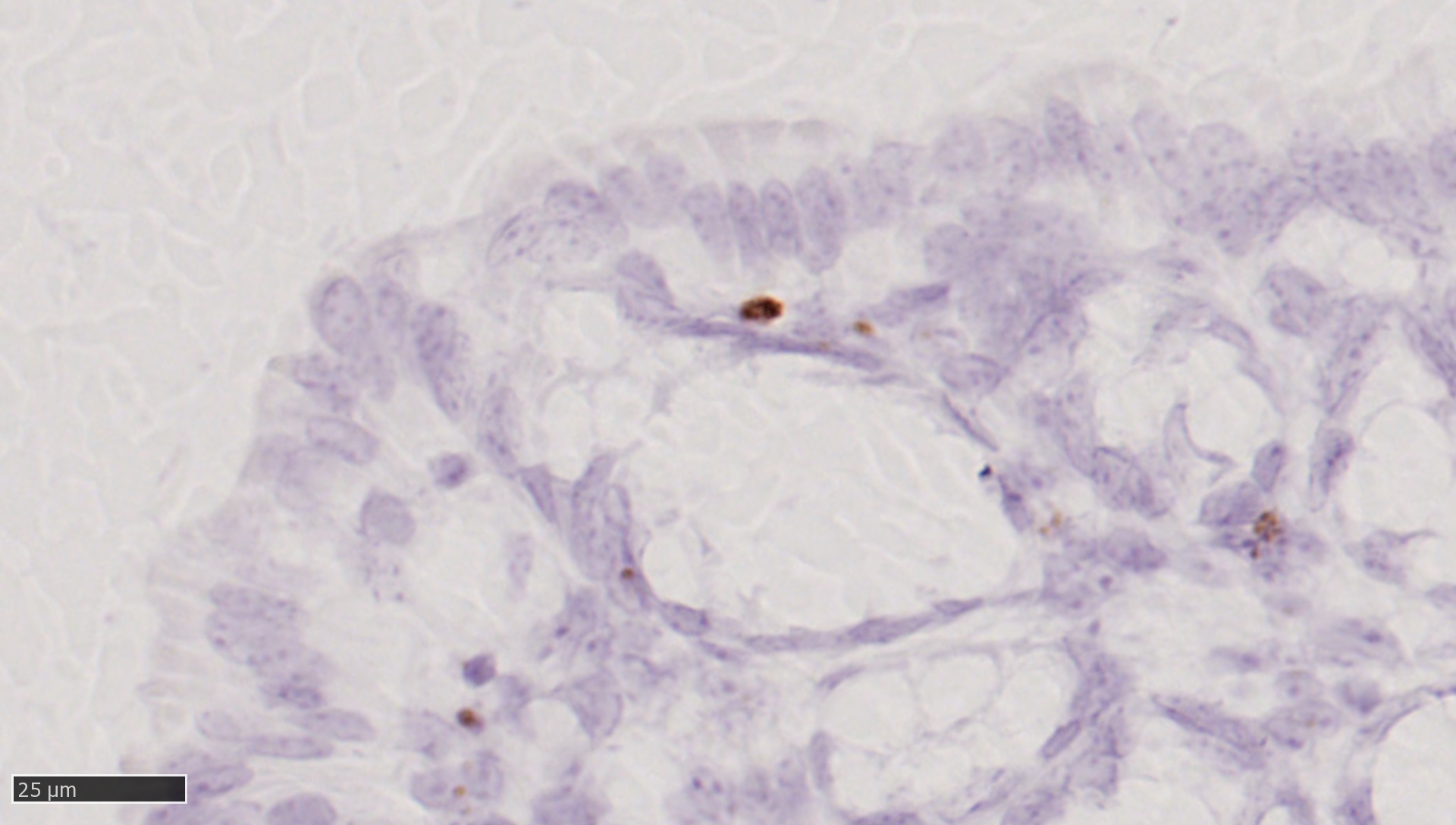

Supplement: Supplementary file 5 — Source Data Fig. 4 [file 44319_2023_13_MOESM5_ESM.zip › Figure 4/4B/zoom col prox Vil1Cre+ 78FxFx.tif]

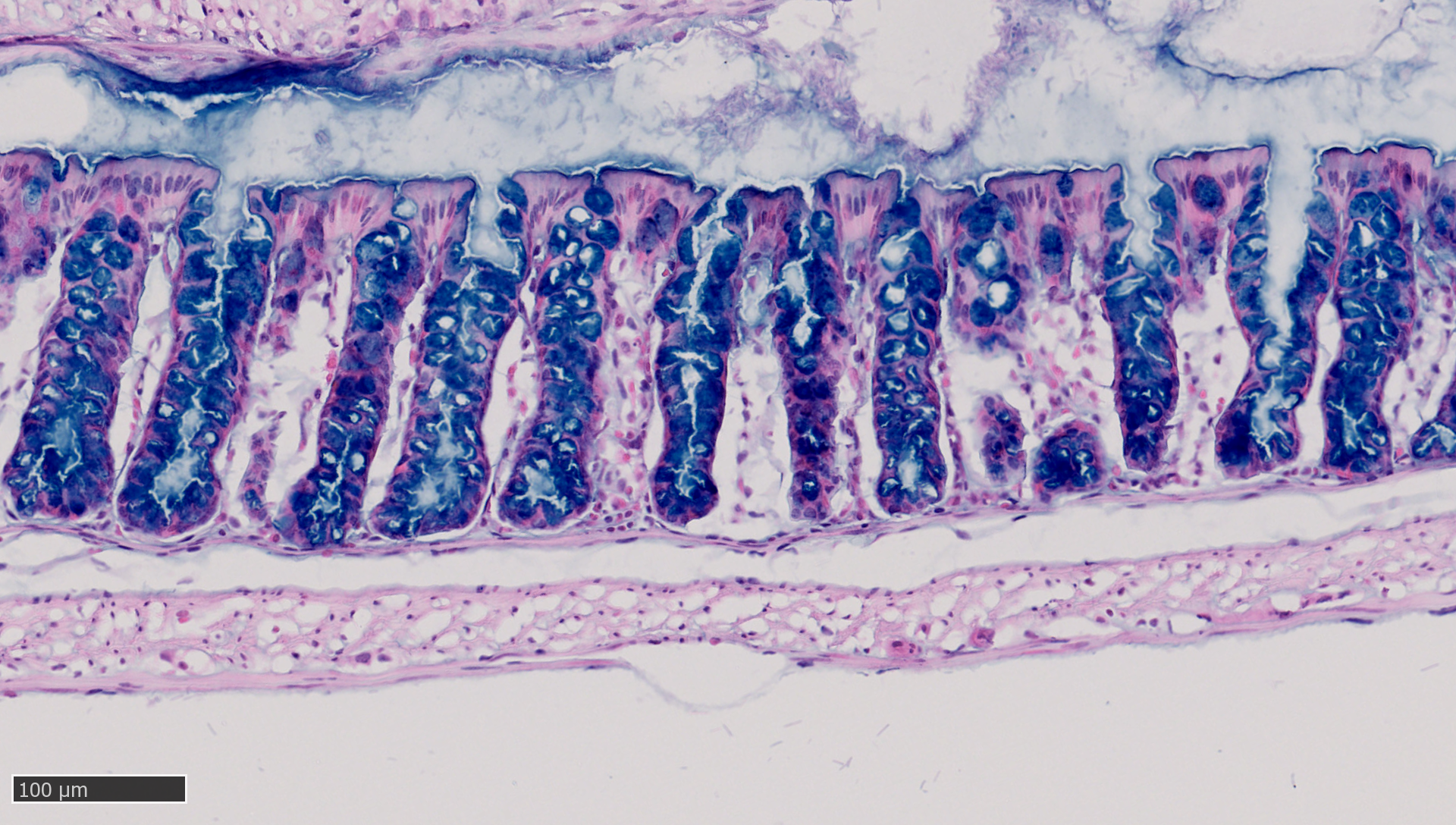

Supplement: Supplementary file 5 — Source Data Fig. 4 [file 44319_2023_13_MOESM5_ESM.zip › Figure 4/4D/Col dist Vil1Cre+ 78FxFx.tif]

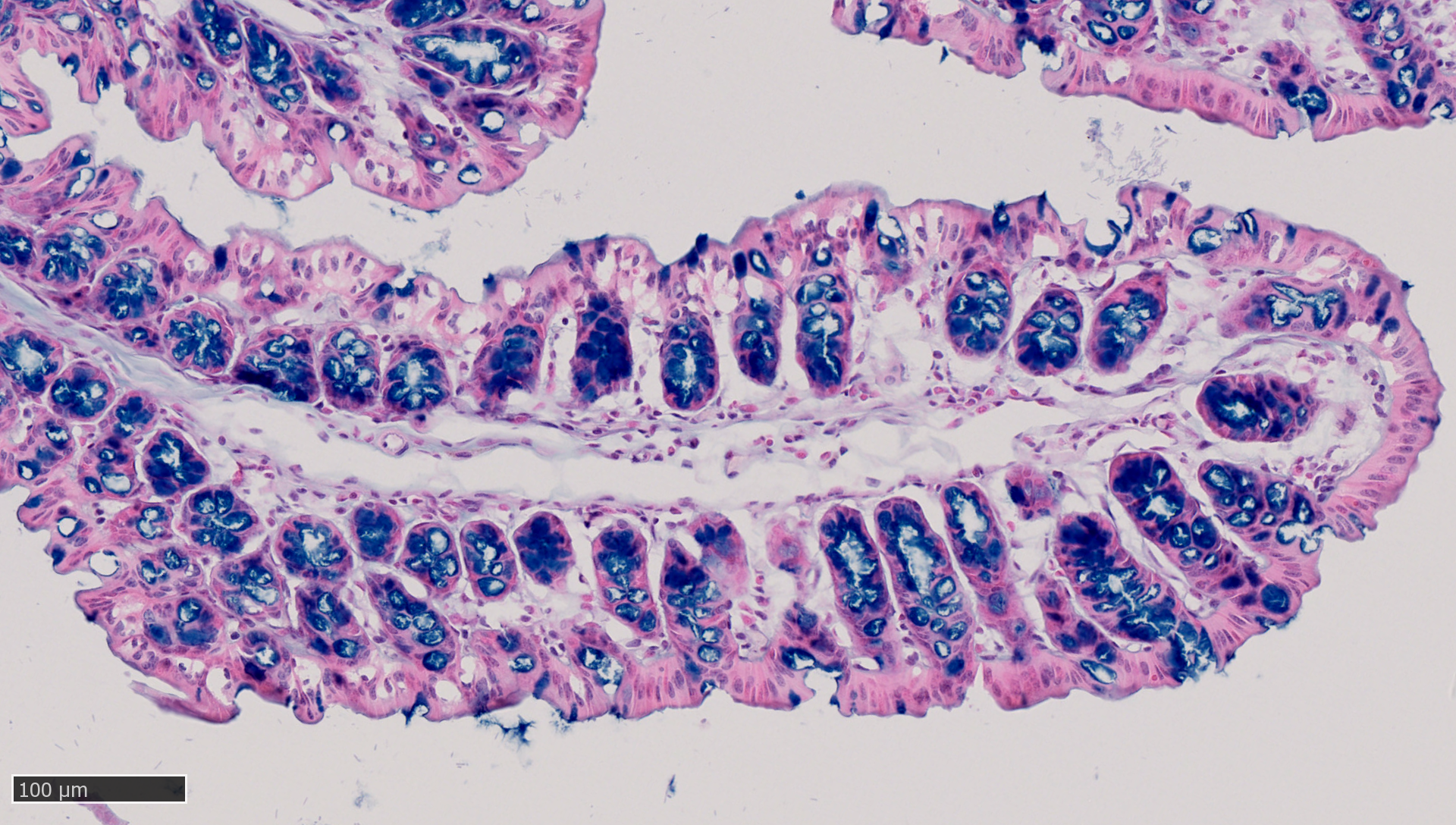

Supplement: Supplementary file 5 — Source Data Fig. 4 [file 44319_2023_13_MOESM5_ESM.zip › Figure 4/4D/Col prox Vil1Cre+ 78++.tif]

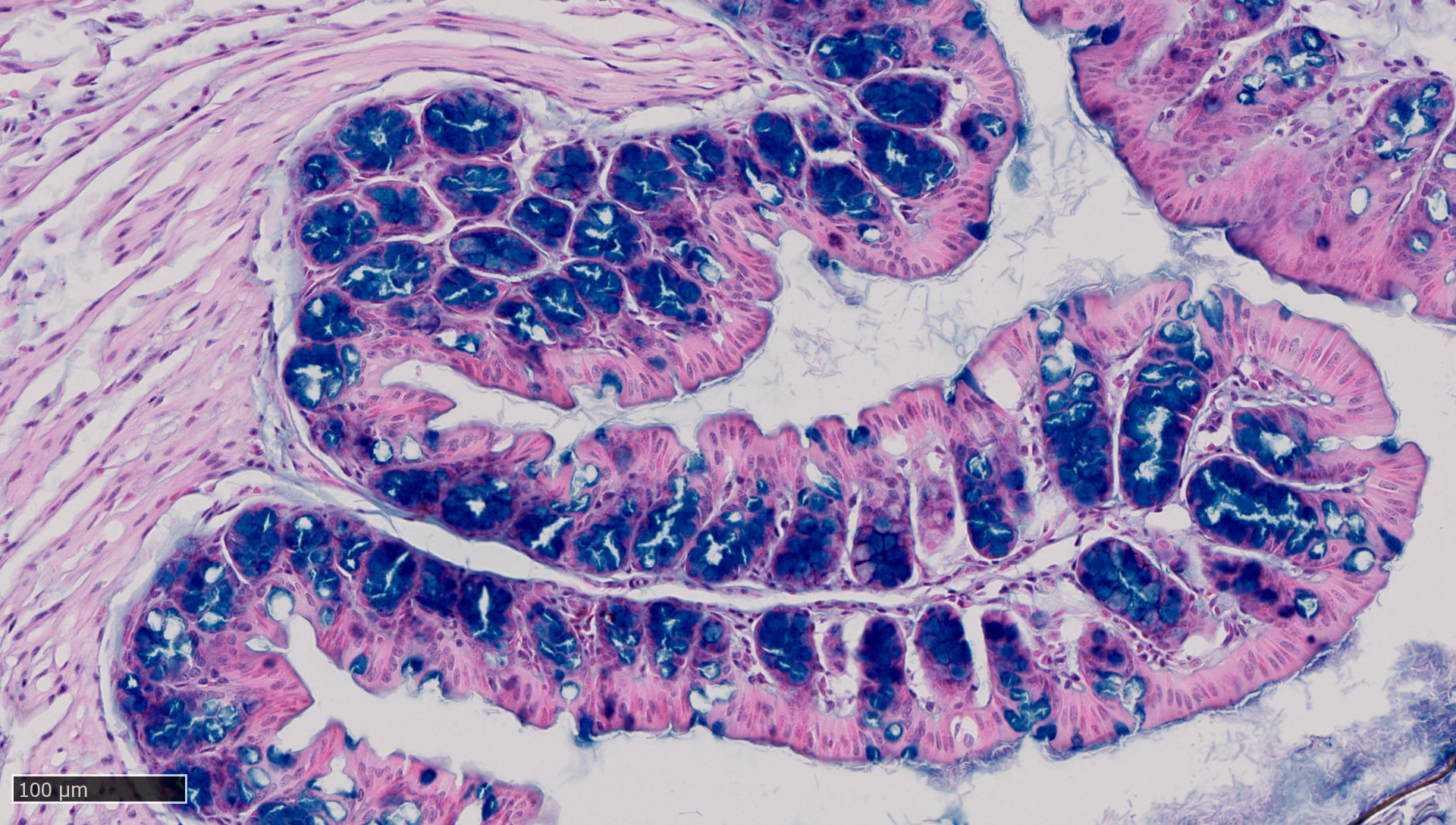

Supplement: Supplementary file 5 — Source Data Fig. 4 [file 44319_2023_13_MOESM5_ESM.zip › Figure 4/4D/Col prox Vil1Cre+ 78FxFx.tif]

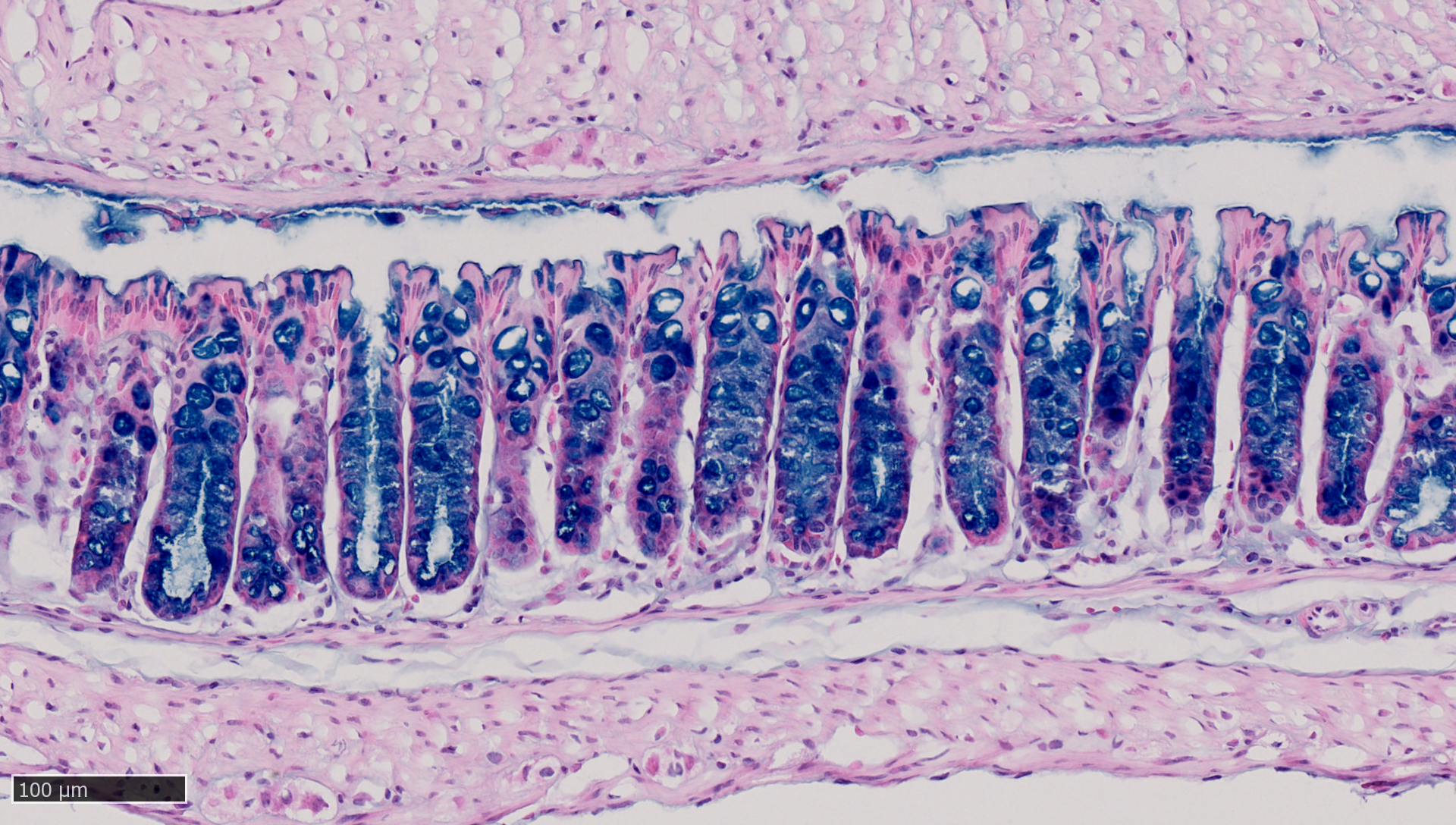

Supplement: Supplementary file 5 — Source Data Fig. 4 [file 44319_2023_13_MOESM5_ESM.zip › Figure 4/4D/low view col dist Vil1Cre+ 78++.tif]

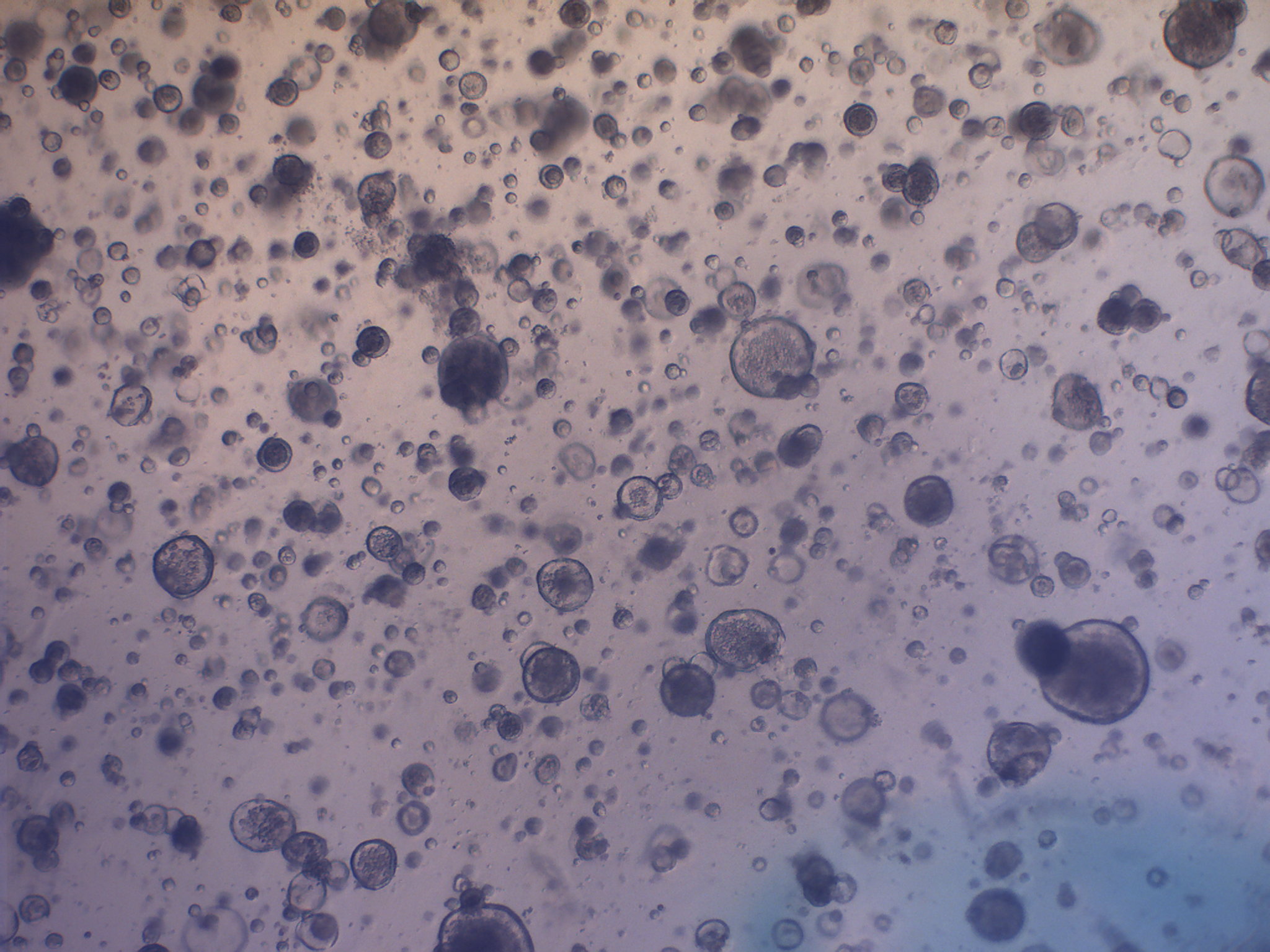

Supplement: Supplementary file 5 — Source Data Fig. 4 [file 44319_2023_13_MOESM5_ESM.zip › Figure 4/4H/m10x8 acet.bmp]

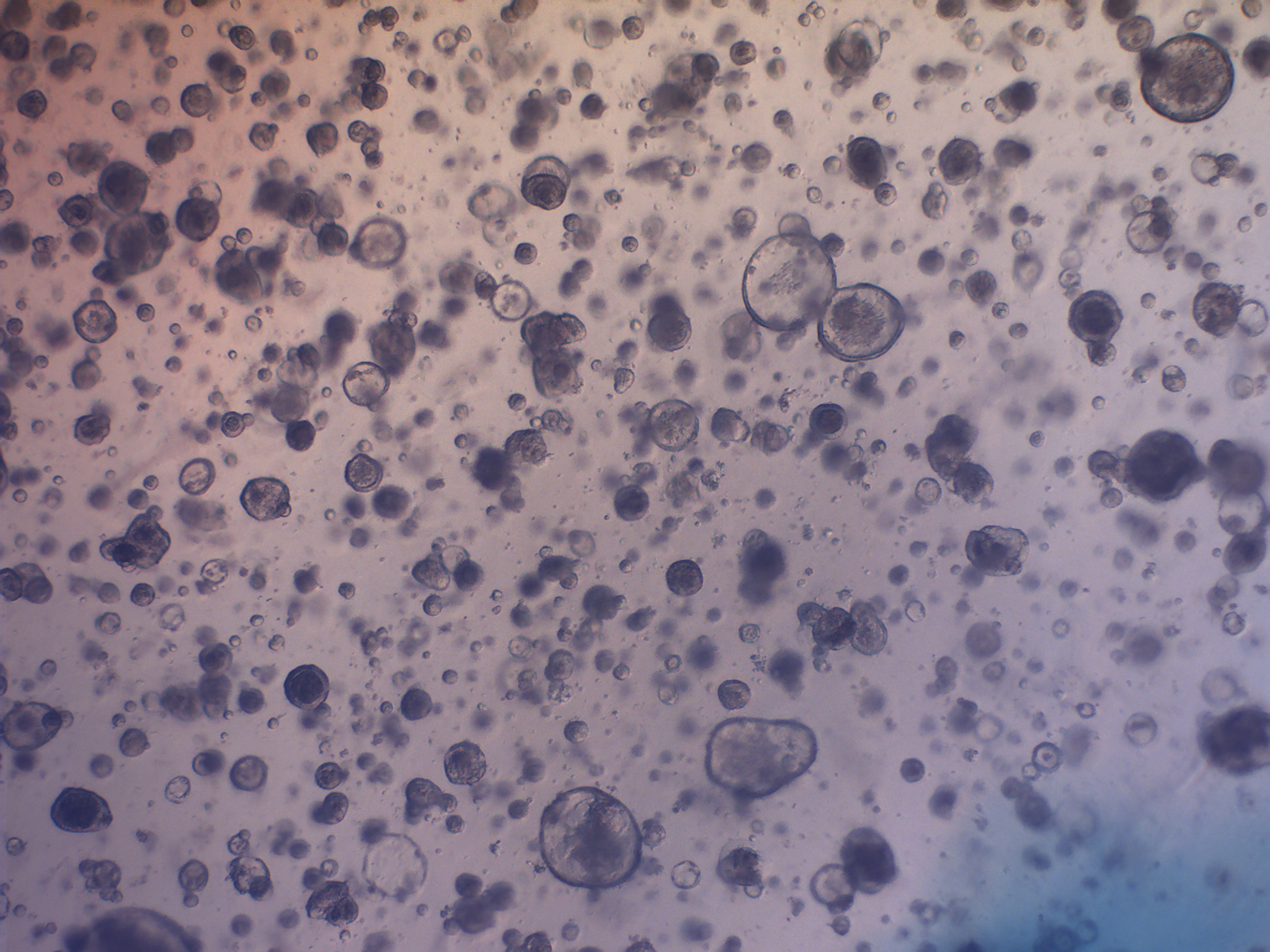

Supplement: Supplementary file 5 — Source Data Fig. 4 [file 44319_2023_13_MOESM5_ESM.zip › Figure 4/4H/m10x8 ctrl.bmp]

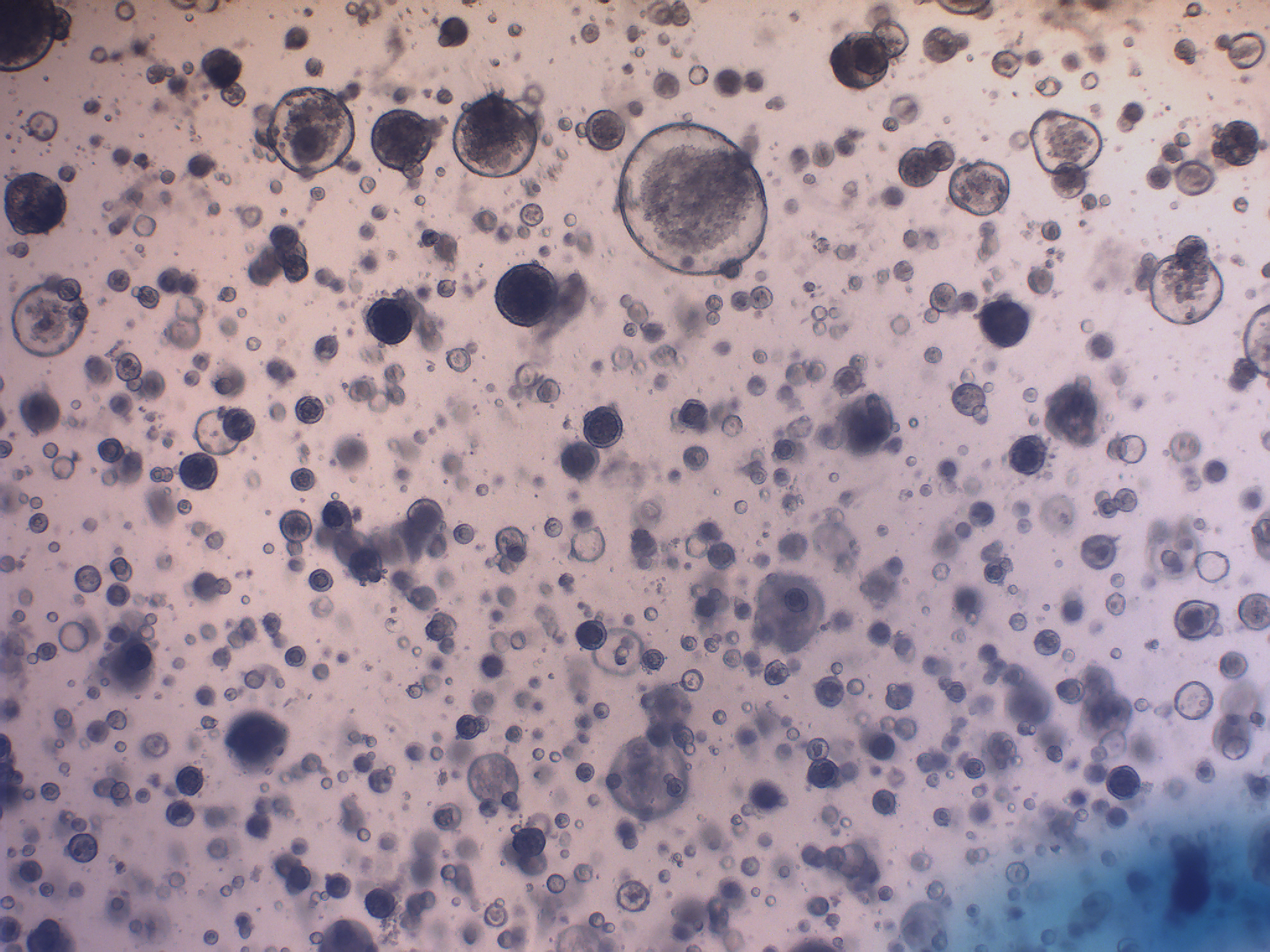

Supplement: Supplementary file 5 — Source Data Fig. 4 [file 44319_2023_13_MOESM5_ESM.zip › Figure 4/4H/m10x8 prop.bmp]

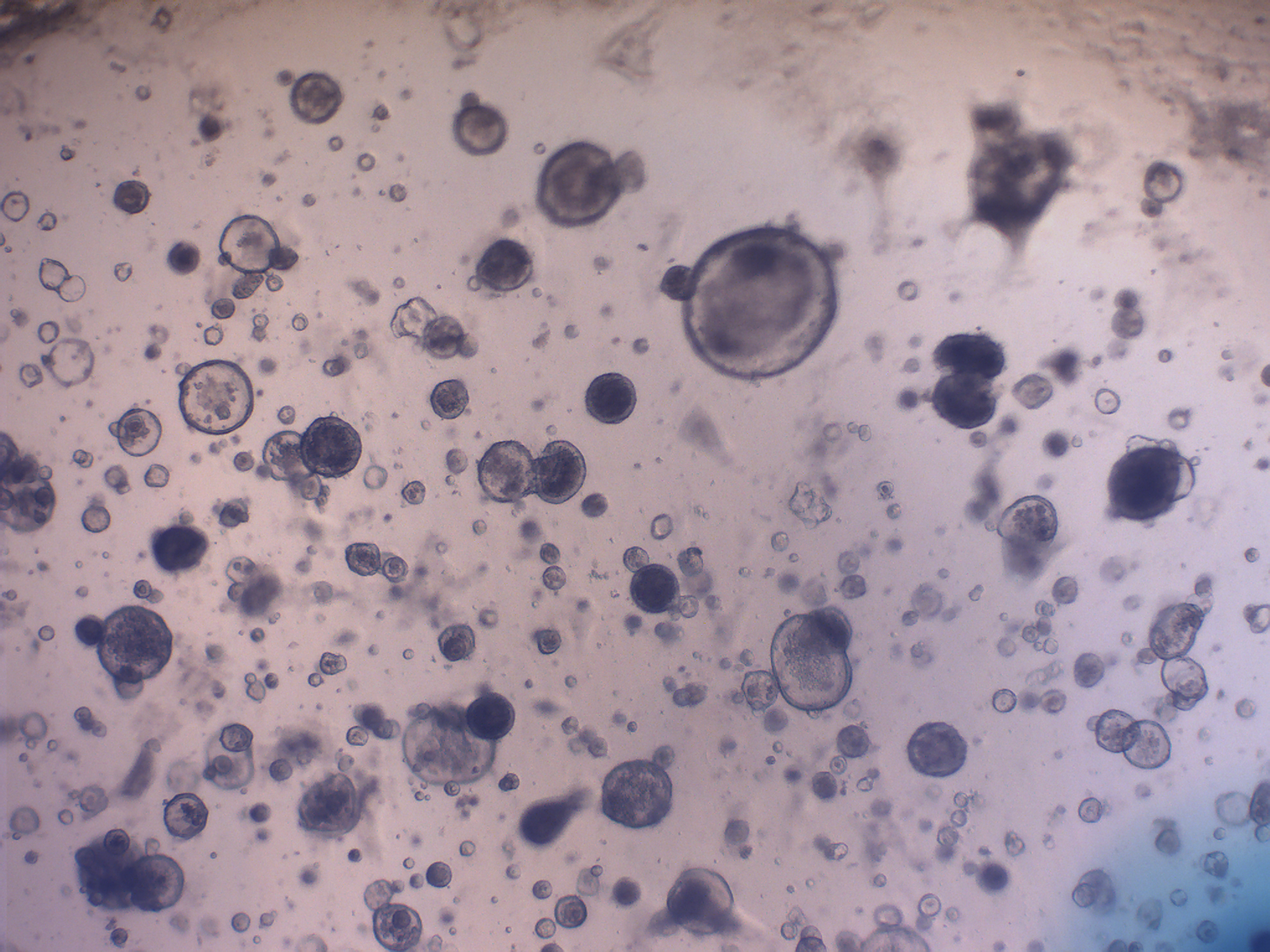

Supplement: Supplementary file 5 — Source Data Fig. 4 [file 44319_2023_13_MOESM5_ESM.zip › Figure 4/4H/M3x3 acet.bmp]

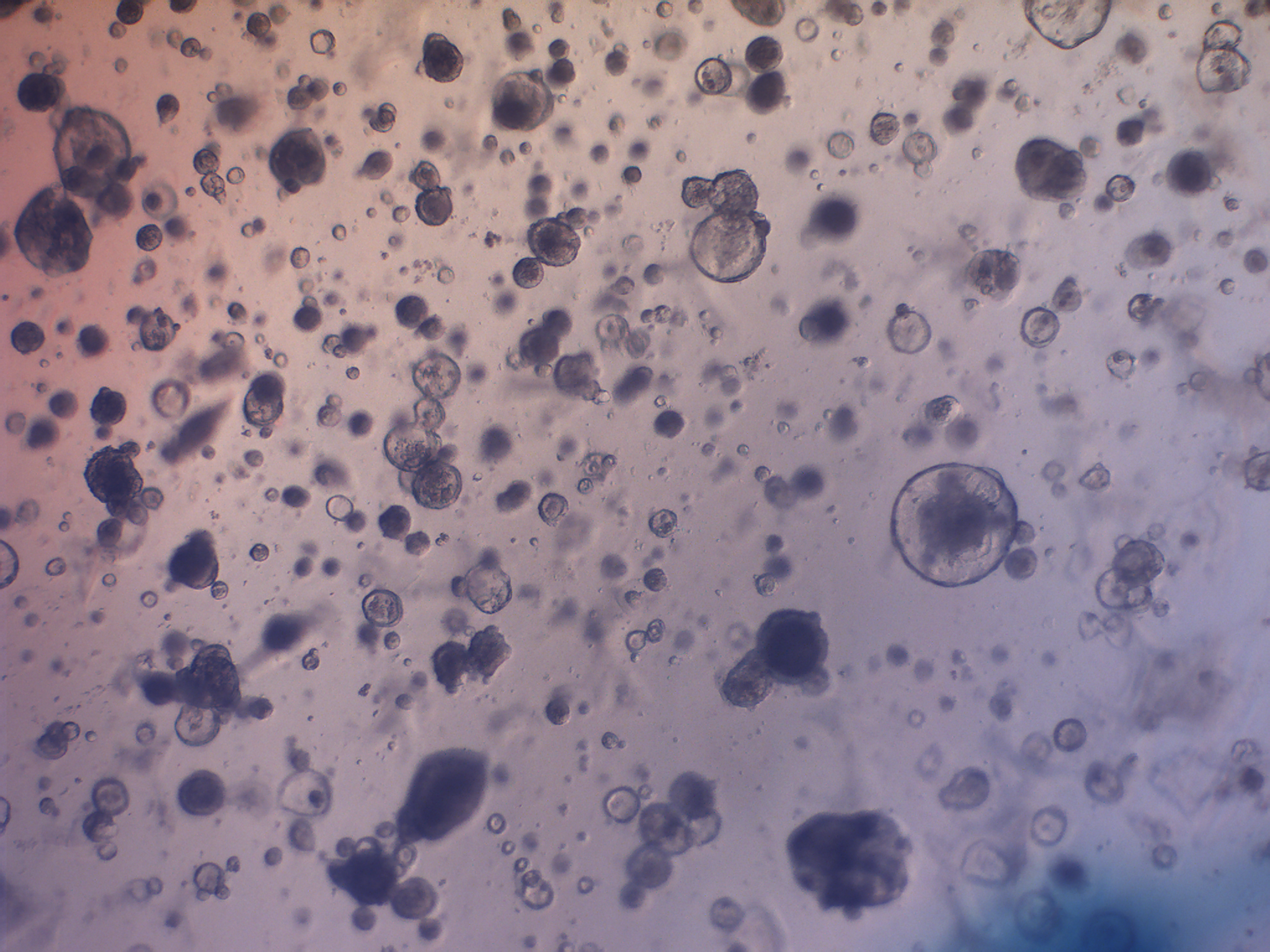

Supplement: Supplementary file 5 — Source Data Fig. 4 [file 44319_2023_13_MOESM5_ESM.zip › Figure 4/4H/M3x3 ctrl.bmp]

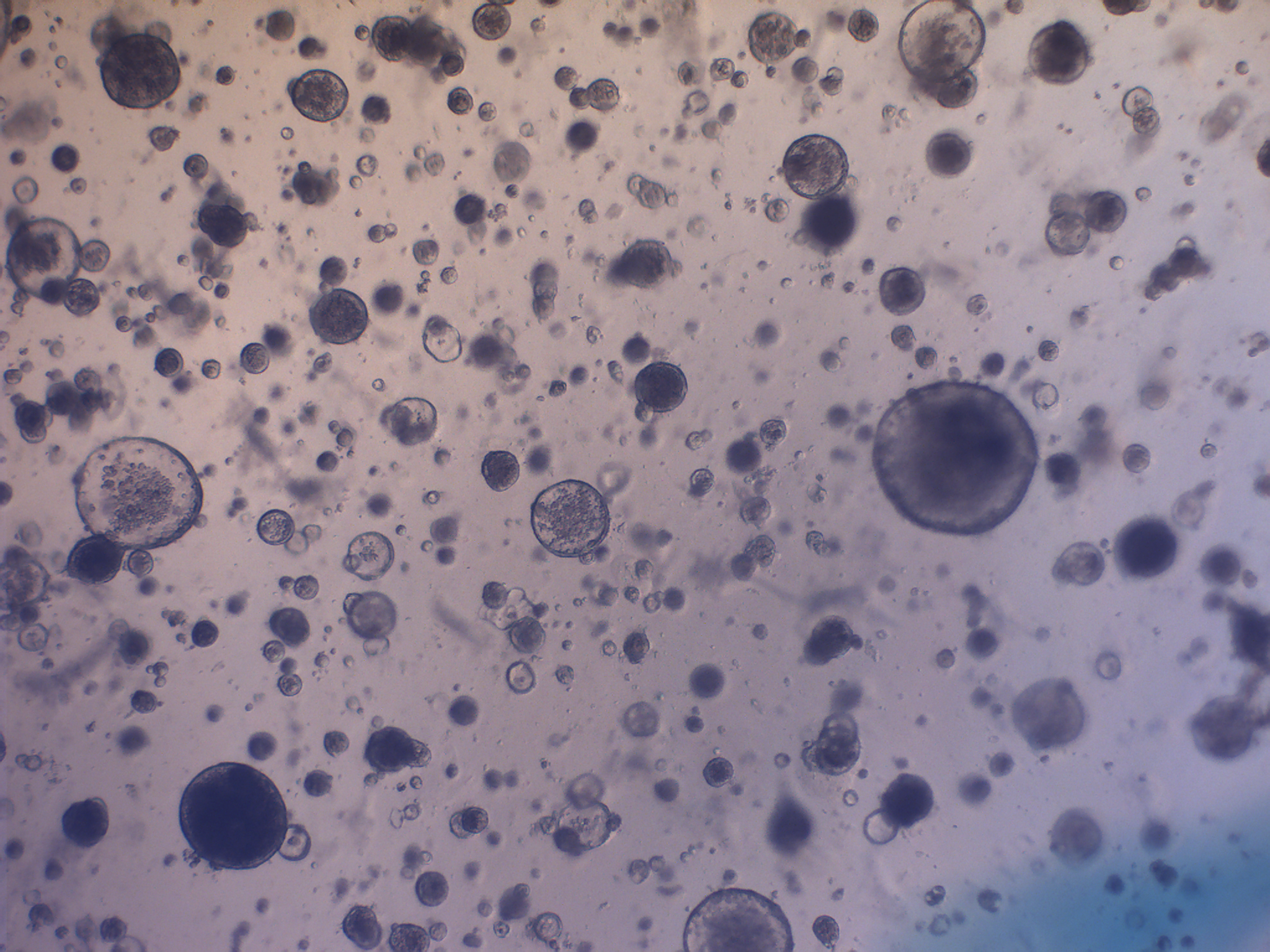

Supplement: Supplementary file 5 — Source Data Fig. 4 [file 44319_2023_13_MOESM5_ESM.zip › Figure 4/4H/M3x3 prop.bmp]
