# Supplementary material for: The olfactory receptor Olfr78 promotes differentiation of enterochromaffin cells in the mouse colon
Source: EMBO Rep. 2023 Dec 15;25(1):19. doi: 10.1038/s44319-023-00013-5 (PMC10897383; doi:10.1038/s44319-023-00013-5)
Supplement: Supplementary file 7 — Expanded View Figures [file 44319_2023_13_MOESM7_ESM.pdf]

Expanded View Figures

Figure EV1. SCFA receptors exhibit unique expression profiles along the small intestine and colon.

(**A**) Representative RNAscope pictures of mesenchymal expression of Olfr78 in the gut. (**B**) Representative RNAscope pictures of mesenchymal expression of Olfr558 in the colon. (**C**) Representative RNAscope pictures of Ffar3 expression in myenteric plexuses in the gut. (**D**) Representative RNAscope pictures of mesenchymal expression of Ffar2 in the Ileum. Data information: Scale bars: 100 μm (low views) or 25 μm (insets). Arrowheads identify isolated SCFA-expressing cells.

© The Author(s)

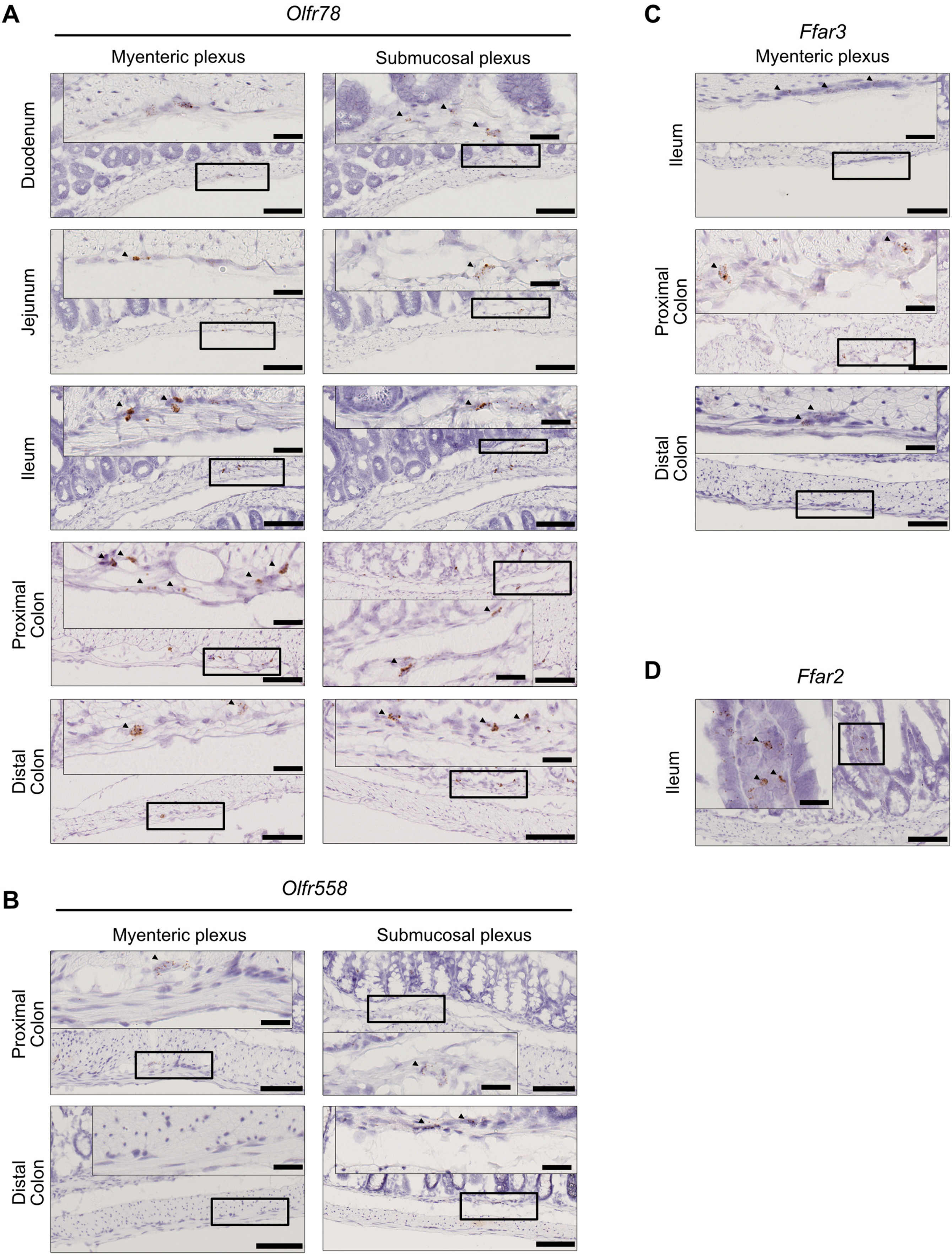
A
Olfr78
Myenteric plexus
Submucosal plexus
Duodenum
Jejunum
Ileum
Proximal Colon
Distal Colon
B
Olfr558
Myenteric plexus
Submucosal plexus
Proximal Colon
Distal Colon
C
Ffar3
Myenteric plexus
Ileum
Proximal Colon
Distal Colon
D
Ffar2
Ileum

 © The Author(s)

A

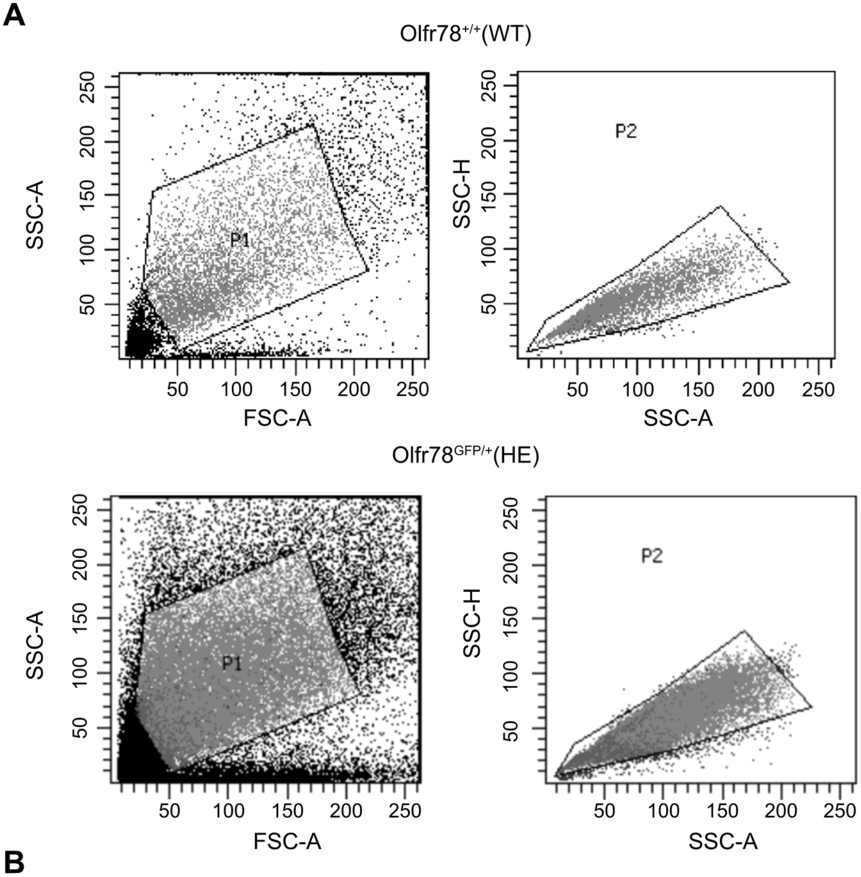

B

Gene	Log2FC	FDR
**Up**		
Amigo2	9.15848307	1.11E-114
Slc18a1	6.79241159	1.67E-103
Celf3	7.61408531	2.95E-100
Tph1	6.61902534	9.38E-98
Cpe	6.44741623	2.23E-94
Chga	6.36548722	1.77E-91
Man1c1	6.74444704	1.58E-87
Map1b	6.49923649	2.73E-87
Neurod1	6.84702226	7.93E-85
Kif1a	6.01803633	7.50E-83
Chgb	6.20893923	1.07E-82
1810006J02 Rik	7.14903542	2.40E-82
Wif1	6.76009617	3.47E-80
Ptprn	6.37906213	2.98E-78
Gnao1	6.34003023	1.25E-77
Cerkl	6.96501199	5.09E-77
Runx1t1	6.95662951	5.95E-75
Rimbp2	5.56144189	3.18E-73
Pcsk1n	7.34781804	2.14E-72
Slc38a11	5.71212377	1.96E-70
**Down**		
Lpo	-4.08743095	2.35E-51
mt-Atp8	-3.96886994	4.88E-43
mt-Atp6	-3.51520681	8.00E-35
mt-Co2	-3.27504437	1.36E-28
Nov	-2.91734831	1.18E-26
Klk1	-2.84714055	2.51E-26
Gm28439	-3.05479056	4.89E-24
Spink4	-2.80673265	3.79E-20
Tg	-11.6632431	3.27E-19
Acpp	-5.45734642	7.55E-19
Sult1c2	-3.42806109	2.31E-17
Gm10222	-2.80082698	4.17E-17
Hells	-2.87316541	1.38E-16
Hmgb2	-2.40677088	2.46E-16
mt-Co3	-2.55896806	3.03E-16
Krt14	-4.37611909	3.99E-16
Mptx1	-2.29087238	4.37E-16
Edn1	-3.28661953	6.56E-16
Sval1	-3.3470063	1.72E-15
Gm1123	-2.59013523	5.57E-15

C

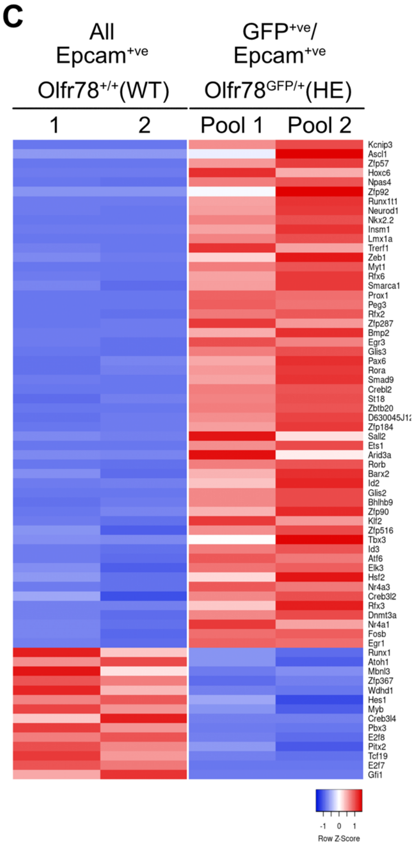

Figure EV2. Olfr78 is expressed in different subtypes of enteroendocrine cells in the colon.

(**A**) FACS strategy for initial population selection and doublets exclusion. (**B**) List of the 20 most up or downregulated genes in Epcam^{+ve}/GFP^{+ve} cells as compared to all Epcam^{+ve} cells, ranked by FDR. (**C**) List of significantly up and downregulated transcription factors in Epcam^{+ve}/GFP^{+ve} cells as compared to all Epcam^{+ve} cells.

© The Author(s)

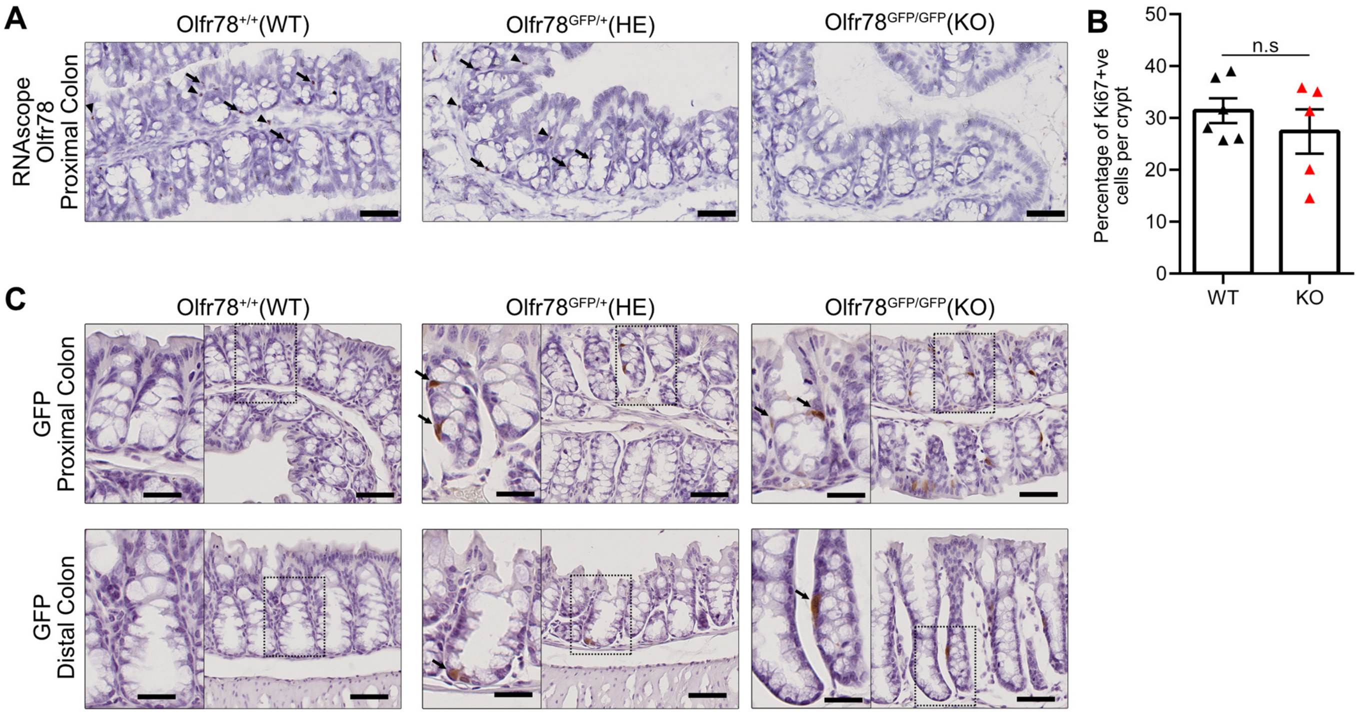

D

Gene Name	Log2 FC
Nrg1	-2.125325
Kcnip3	-2.11699146
Rab6b	-2.0107952
Rab3a	-1.96819794
Cacnb1	-1.94381716
Baiap2	-1.80866151
Fxyd6	-1.79893723
P2ry4	-1.64636305
Hap1	-1.57458335
Rab33b	-1.51457317
Clstn2	-1.5033362
Smo	-1.48509329
Chrnb2	-1.47506227
Lrfn4	-1.45700079
Bnip3	-1.38177195
Sh3gl1	-1.32638774
Hcn4	-1.3238633
Dbn1	-1.28762639
Mapk8ip2	-1.23274218
Sh2d5	-1.18957224
Ddc	-1.16050341
Ache	-1.15647561
Unc13a	-1.14981989
Il1r1	-1.1477221
Ptprn	-1.13272638
Kcnj2	-1.13103403
Rab3c	-1.11624983
Asic2	-1.09160195
Nefm	-1.03936243
Camk2b	-1.02363123
Cdk5	-1.02197837
Synpo	-1.01534796
Pacsin1	-1.01449957
Cacna1h	-0.99539749
Cbln1	-0.99008453
Shisa7	-0.98845838
Prkaca	-0.97575245
Pcsk1	-0.97528142
Syp	-0.97449372
Slc6a7	-0.93161303
Stxbp1	-0.92880439

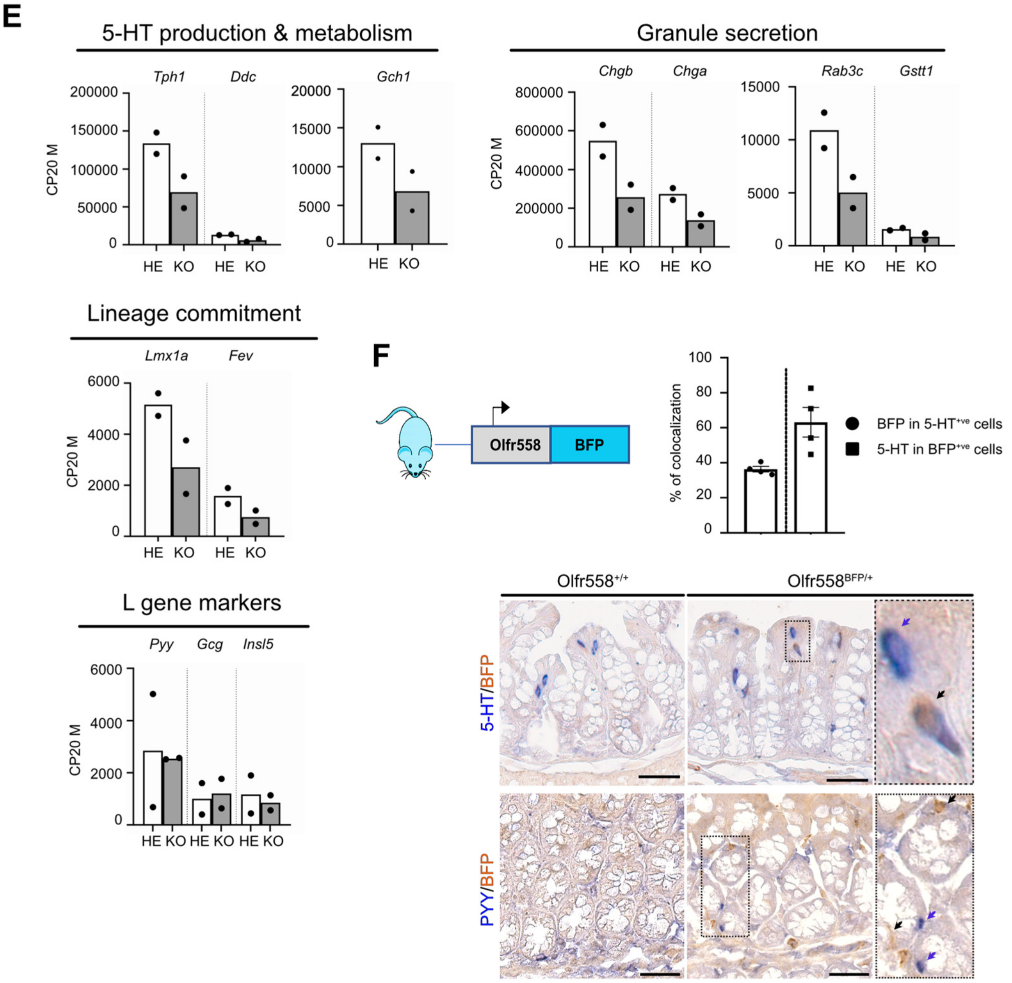

Figure EV3. Loss of Olfr78 impairs terminal differentiation into enterochromaffin cells.

(**A**) Olfr78 expression in proximal colon of WT, HE and Olfr78-GFP KO mice analyzed by RNAscope. Arrows and arrowheads identify epithelial and mesenchymal Olfr78-expressing cells, respectively. (**B**) Quantification of cell proliferation (Ki67^{+ve} cells) in proximal colon crypts of Olfr78-GFP WT and KO mice. Each symbol indicates the value for a given mouse (n = 6 WT, 5 KO). (**C**) GFP expression in proximal colon of WT, HE and Olfr78-GFP KO mice analyzed by Immunohistochemistry. (**D**) List of downregulated genes in Olfr78-GFP KO Epcam^{+ve}/GFP^{+ve} cells related to GSEA pre- or post-synapse gene lists, ranked by Log_2(Fold Change). (**E**) Histograms showing the expression of target genes in the bulk RNAseq from Fig. 3D. CP20M: counts per 20 million mapped reads. (**F**) Upper panel: Quantification of colocalization between 5-HT or PYY and BFP in the newly generated Olfr558-BFP mouse line (BFP cassette replacing the Olfr558 coding region). Each symbol indicates the value of a given mouse ($n = 4$). Lower panel: Representative pictures of double immunohistochemistry showing 5-HT/BFP and PYY/BFP co-stainings in proximal colon of Olfr558-BFP WT or HE mice. Data information: Scale bars: 50 µm (**A**, **C** and **F**) or 25 µm (**A** and **C**, inset). Data are represented as mean ± SEM.; unpaired t-test, n.s = not significant.

 © The Author(s)

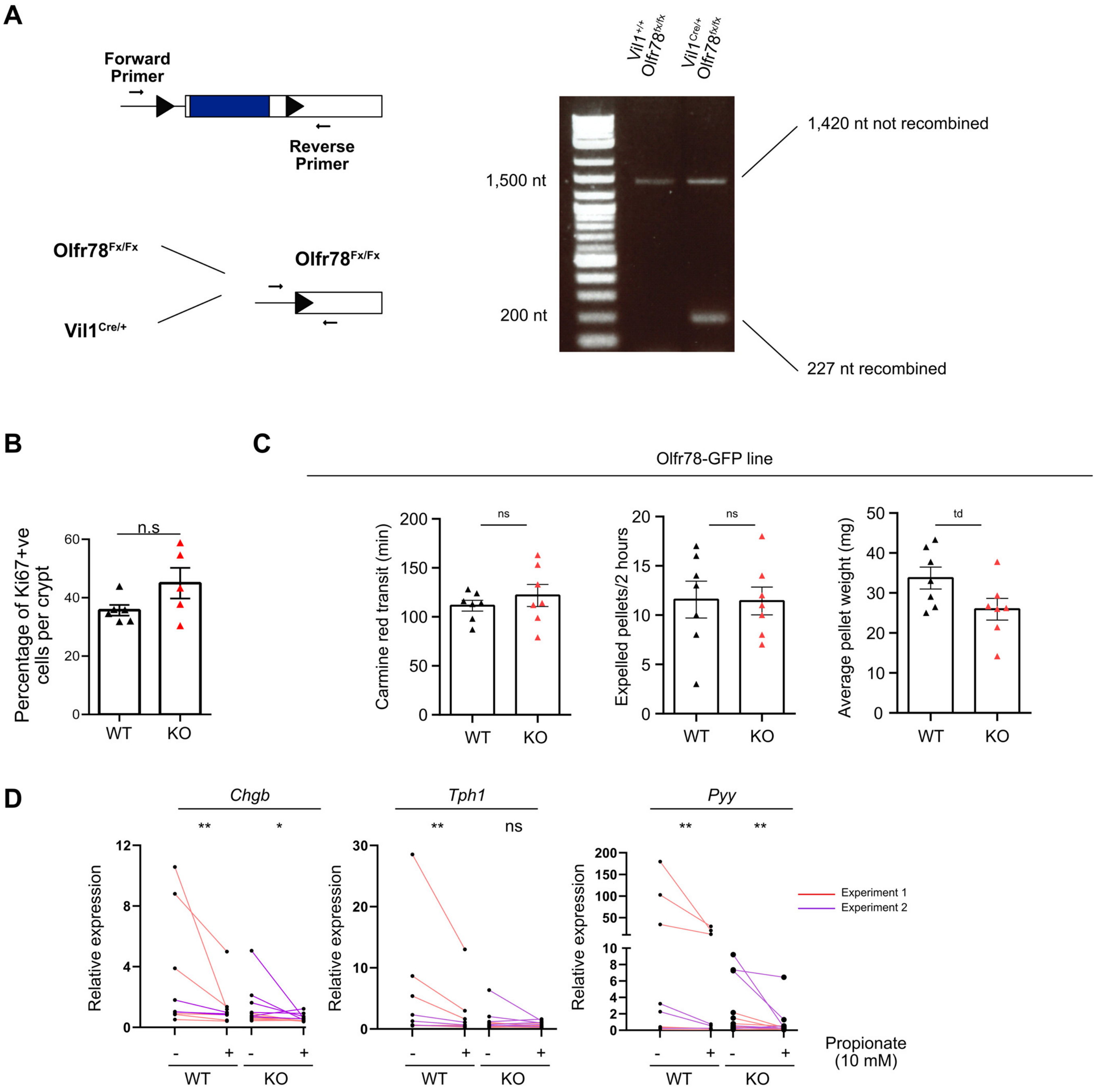

E

Olfr558 Ct values	Mean ± sem (minimum-maximum)	
	Untreated (5)	Acetate 20 mM (5)
Olfr78+/+	30.55 ± 1.178 (26.38-32.53)	30.20 ± 1.123 (25.87-32.11)
Olfr78GFP/GFP	30.41 ± 1.242 (26.84-33.19)	32.27 ± 0.139 (31.80-32.63)

Gcg Ct values	Mean ± sem (minimum-maximum)	
	Untreated (6)	Acetate 20 mM (6)
Olfr78+/+	30.05 ± 1.494 (26.6-35.0)	30.05 ± 1.550 (25.2-33.8)
Olfr78GFP/GFP	35.58 ± 0.971 (32.1-37.8)	35.32 ± 0.661 (32.9-37.4)

© The Author(s)

Figure EV4. Terminal differentiation into serotonin-producing cells is regulated by epithelial Olfr78 expression.

(**A**) Left: PCR strategy for loxp sites recombination verification in Vil1$^{Cre/+}$-Olfr78$^{fx/fx}$. Right: Gel electrophoresis showing WT and recombinant bands in Vil1$^{Cre/+}$-Olfr78$^{fx/fx}$. (**B**) Quantification of Ki67^{+ve} cells in proximal colon crypts of Olfr78-GFP WT and KO mice. Each symbol indicates the value for a given mouse. (**C**) Histograms showing the transit time of carmine red (left), the number of fecal pellets expelled during 2 h (middle) and the average wet stools weight (right) in Olfr78-GFP WT or KO mice. Each symbol indicates the value for a given mouse ($n = 7$ WT and 7 KO). (**D**) Graphs showing the relative expression levels of EEC markers analyzed by qRT-PCR on colon organoids after 48 h of treatment with propionate at 10 mM. Data are reported as the relative expression levels in treated and control conditions in Olfr78-GFP WT and KO organoids. Each symbol indicates the individual value of a given organoid line in each experiment ($n = 3$–6 WT and 6 KO). Colored lines identify paired samples in 2 independent experiments. (**E**) Raw cycle threshold values obtained from qPCR experiments performed on Olfr78-GFP WT or KO organoids (mean ± SEM). Data information: Data are represented as mean ± SEM.; (**B**, **C**) unpaired t-test n.s = not significant; td: tendency ($p = 0.0673$), (**D**) Wilcoxon matched-pairs signed rank test, n.s = not significant, *$P < 0.05$, **$P < 0.01$.

 © The Author(s)

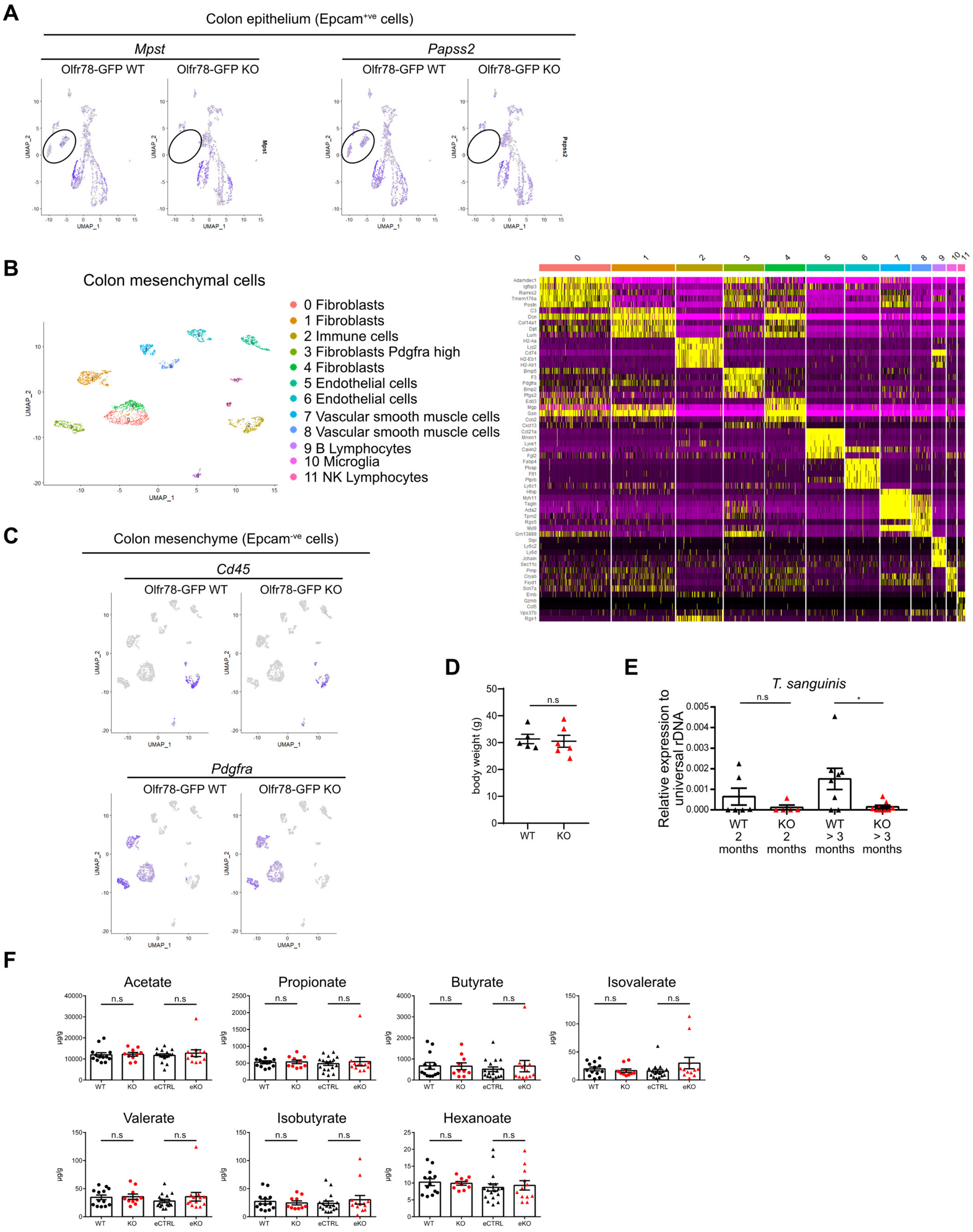
A
Colon epithelium (Epcam+ve cells)
Mpst
Papss2
Olfr78-GFP WT
Olfr78-GFP KO
UMAP_1
UMAP_2
B
Colon mesenchymal cells
0 Fibroblasts
1 Fibroblasts
2 Immune cells
3 Fibroblasts Pdgfra high
4 Fibroblasts
5 Endothelial cells
6 Endothelial cells
7 Vascular smooth muscle cells
8 Vascular smooth muscle cells
9 B Lymphocytes
10 Microglia
11 NK Lymphocytes
C
Colon mesenchyme (Epcam-ve cells)
Cd45
Pdgfra
Olfr78-GFP WT
Olfr78-GFP KO
D
body weight (g)
n.s
WT
KO
E
T. sanguinis
Relative expression to universal rDNA
n.s
*
WT 2 months
KO 2 months
WT > 3 months
KO > 3 months
F
Acetate
Propionate
Butyrate
Isovalerate
Valerate
Isobutyrate
Hexanoate
μg/g
n.s
WT
KO
eCTRL
eKO

Figure EV5. Loss of Olfr78 expression alters colon homeostasis.

(**A**) Umap of Olfr78-GFP WT and KO colon epithelial cells showing *Mpst or Papss2* expression. Circles identify enriched clusters differentially present in WT and KO mice. (**B**) Left panel: Merged UMAP of colon mesenchymal cells from Olfr78-GFP WT and Olfr78-GFP KO mice. Right panel: Heatmap showing the top 5 markers of each cluster of the UMAP. (**C**) Umap of Olfr78-GFP WT and KO colon mesenchymal cells showing *Cd45 or Pdgfra* expression. (**D**) Weight of adult Olfr78-GFP mice. Each symbol indicates the value for a given mouse ($n = 5$ WT and 6 KO). (**E**) Analysis of *Turicibacter sanguinis* prevalence by qPCR in the fecal microbiota of Olfr78-GFP WT or KO mice at different ages. Each symbol indicates the value for a given mouse ($n = 6$–8 WT and 5–9 KO). (**F**) Quantification of fecal SCFA concentrations. Each symbol indicates the value for a given mouse (Olfr78-GFP line: $n = 13$ WT and 10 KO; VilCre/Olfr78-Fx line: $n = 17$ controls and 12 eKO). Data information: Data are represented as mean ± SEM.; (**D**, **E**, **F**) Mann–Whitney tests, (**E**), n.s = not significant; *$P < 0.05$.

 © The Author(s)
